# (RPh_3_P)[Mn(dca)_3_]: A Family of Glass-Forming Hybrid Organic–Inorganic Materials

**DOI:** 10.1021/acs.inorgchem.4c04181

**Published:** 2024-12-19

**Authors:** Bikash Kumar Shaw, Lucia Corti, Joshua M. Tuffnell, Celia Castillo-Blas, Patrick Schlachta, Georgina P. Robertson, Lauren McHugh, Adam F. Sapnik, Sebastian A. Hallweger, Philip A. Chater, Gregor Kieslich, David A. Keen, Sian E. Dutton, Frédéric Blanc, Thomas D. Bennett

**Affiliations:** † Department of Materials Science and Metallurgy, University of Cambridge, Cambridge CB3 0FS, U.K.; ‡ Department of Chemistry, Technical University of Munich, 85748 Garching, Germany; § Department of Chemistry, University of Liverpool, Crown Street, Liverpool L69 7ZD, U.K.; ∥ Leverhulme Research Centre for Functional Materials Design, Materials Innovation Factory, University of Liverpool, Liverpool L7 3NY, U.K.; ⊥ Department of Physics, University of Cambridge, Cambridge CB3 0FS, U.K.; # Diamond Light Source Ltd., Diamond House, Harwell Campus, Didcot OX11 0DE, Oxfordshire, U.K.; ∇ ISIS Facility, Rutherford Appleton Laboratory, Harwell Campus, Didcot OX11 0QX, U.K.; ○ Stephenson Institute for Renewable Energy, University of Liverpool, Crown Street, Liverpool L69 7ZF, U.K.

## Abstract

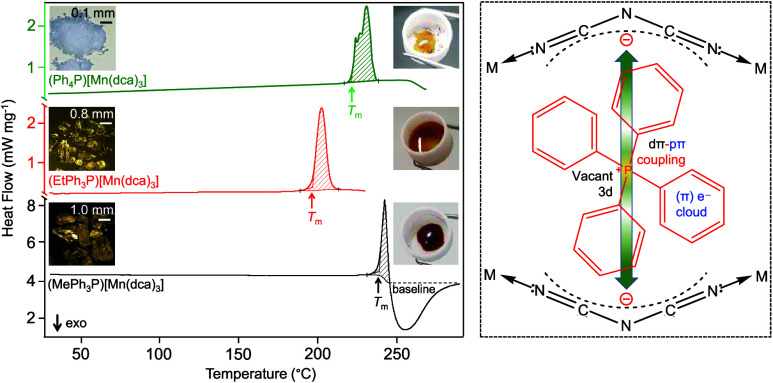

ABX_3_-type hybrid organic–inorganic structures have recently emerged as a new class of meltable materials. Here, by the use of phenylphosphonium derivatives as A cation, we study liquid- and glass-forming behavior of a new family of hybrid structures, (RPh_3_P)[Mn(dca)_3_] (R = Me, Et, Ph; dca = dicyanamide). These new compounds melt at 196–237 °C (*T*_m_) and then vitrify upon cooling to room temperature, forming glasses. *In situ* glass formation of this new family of materials was probed on a large scale using a variable-temperature PXRD experiment. Structure analyses of the crystalline and the glasses were carried out by solid-state nuclear magnetic resonance spectroscopy and synchrotron X-ray total scattering techniques for using the pair distribution function. The mechanical properties of the glasses produced were evaluated showing promising durability. Thermal and electrical conductivities showed low thermal conductivities (κ ∼ 0.07–0.09 W m^–1^ K^–1^) and moderate electrical conductivities (σ ∼ 10^–4^–10^–6^ S m^–1^) at room temperature, suggesting that by the precise control of the A cation, we can tune meltable hybrid structures from moderate conductors to efficient thermal insulators. Our results raise attention on the practical use of this new hybrid material in applications including, e.g., photovoltaic devices to prevent light-deposited heat (owing to low κ_RT_), energy harvesting thermoelectric, etc., and advance the structure–property understanding.

## Abstract 2

Design of melt-quench glasses from crystalline ABX_3_ hybrid organic−inorganic materials having dπ···pπ coupled phenylphosphonium A cations.

## Introduction

Hybrid organic–inorganic materials occupy a prominent position within solid-state materials due to their functional properties such as ion transport, ferroelectricity, and multiferroicity.^1,2^ Over the past 2 decades, ABX_3_-type hybrid organic–inorganic materials have emerged as a large class of crystalline materials, where [BX_6_] octahedra form pseudocuboctahedra cavities in which they accommodate A cations. In the case of classical ABX_3_ perovskites, A is a cation such as an alkaline earth metal, B is a transition metal, and X is a monatomic anion such as an oxide, halide, or sulfide. In hybrid organic–inorganic perovskites, the A cation is replaced by larger organic cations (such as alkylammonium) and X is specifically a halide, such as 3D MAPbI_3_ (MA = methylammonium) or 2D 1-MeHa_2_PbI_4_ (MeHa = 1-methyl-hexylammonium).^3,4^ Introduction of polydentate anions at the X site, e.g., cyanide [CN^–^], dicyanamide [dca, N(CN)_2_^–^], formate [HCOO^–^] and hypophosphite [H_2_POO^–^], lead to structures (e.g., (TPrA)[Mn(dca)_3_] (TPrA = tetrapropylammonium)) that differ significantly in terms of their physical properties.^1,2,5−8^ Therefore, such materials are often termed molecular perovskites, serving to highlight the use of molecules on the X-site. When choosing A cation too large or too small, other structure ABX_3_ types are formed that we simply referred to as hybrid organic–inorganic networks, see Table 1. Overall, minimal void space and concomitant high structural density provide more stability to these hybrid materials compared to traditional soft porous subfamily like coordination polymers (CPs) and metal–organic frameworks (MOFs), which also leads to multifunctional properties such as dielectric,^9^ ferroelectric,^10^ multiferroic,^11−13^ photovoltaic,^14^ or barocaloric behavior.^8^

**Table 1:** Comparison of Thermo-Structural Properties of Manganese Dicyanamide-Based ABX_3_ Structures with Change in Type of A Site Cations from Alkylammonium (Previous Study)^21^ to Phenylphosphonium (Present Study)

							*a*_g_(A)[Mn(dca)_3_]		
(A)[Mn(dca)_3_]	space group	structure type	*T*_d_ (°C)	*T*_m_ (°C)	Δ*H*_f_ (kJ mol^–1^)	Δ*S*_f_ (J mol^–1^ K^–1^)	*T*_g_ (°C)	*T*_g_/*T*_m_	ref
(TPrA)	*P*4̅2_1_*c*	perovskite	281	262	47	88	223	0.92	(17,21)
(TBuA)	*P2*_1_2_1_2	triple rutile	282	185	59	128	33	0.66	(21)
(TPnA)	*Pnna*	LiSbO_3_	283	149	56	132	9	0.66	(21)
(MePh_3_P)	*P*2_1_2_1_2_1_	triple rutile	288	237	52	101	33	0.60	this work
(EtPh_3_P)	*P*2_1_2_1_2_1_	triple rutile	285	196	52	110	75	0.74	this work
(Ph_4_P)	*P*2/*n*	2D square	308	222	57	115	77	0.71	this work

Despite the dominance of the solid (crystalline) state in the hybrid organic–inorganic ABX_3_ material family, examples of melting are available,^4,15^ particularly in the dicyanamide subfamily.^16,17^ This is unusual since thermal decomposition is typically observed prior to melting in most crystalline CP/MOFs.^18−20^ In our recent studies, we have shown that the (TAlA)[M(dca)_3_] (TAlA = tetralkylammonium, e.g., tetrapropylammonium (TPrA), tetrabutylammonium (TBuA), tetrapentylammonium (TPnA); M = Mn, Fe, Co) material series undergo melting at very low temperatures, *T*_m_ < 250 °C (e.g., the lowest is 106 °C for (TPnA)[Co(dca)_3_]) via M–N coordination bond breaking and the formation of undercoordinated M centers.^17,21^ Interestingly, cooling these high-temperature liquids back to room-temperature results in glass formation. We established a direct correlation between the size of the organic A cation and the melting (*T*_m_) and glass transition (*T*_g_) temperatures in (TAlA)[M(dca)_3_]. In particular, increasing the size of the A cation from TPrA → TBuA → TPnA was found to lower the structural symmetry and *T*_m_ through an increase in the entropy of fusion (Δ*S*_f_). This was due to the increase in alkyl chain lengths among the cations in the solid state. The glasses designed from these hybrid organic–inorganic structures exhibit polymer-like thermomechanical properties and suggest a greater accessibility and processability of the liquid state compared to inorganics and MOF glasses, e.g., zeolitic imidazolate frameworks (ZIFs).^17^ Importantly, the said amorphous hybrid organic–inorganic networks and similar amorphous CP/MOFs were found to exhibit unusual electronic and phononic properties such as high proton conductivity,^22^ metallic charge transport,^23^ high electrical conductivity,^24^ etc. This has several potential applications in fuel cells^22^ and energy conversion thermoelectrics^17,24^ as well as opens up new material directions toward amorphous-phase energy conversion devices.^25−29^

**Figure 1:**
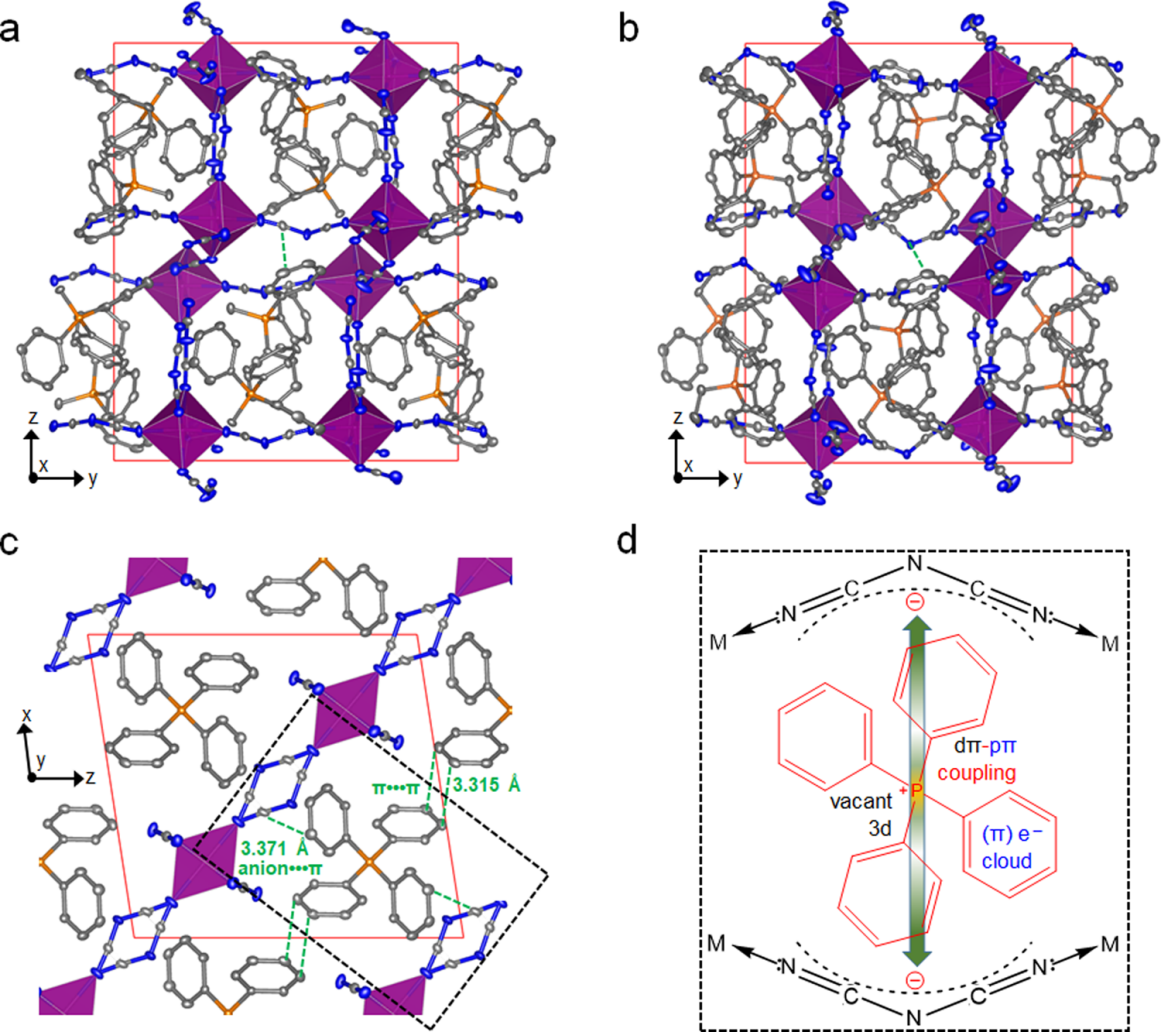
Simplified structure of (a) (MePh_3_P)[Mn(dca)_3_], (b) (EtPh_3_P)[Mn(dca)_3_], and (c) (Ph_4_P)[Mn(dca)_3_] at 298 K.^30,31^ Reproduced from refs (30,31). Copyright [2001, 2004] American Chemical Society. Atom color code: octahedral polyhedra of Mn, purple; P, orange; C, gray; N, blue. All H atoms have been omitted for clarity. Red lines indicate the unit cell. The dashed box (black) in (c) shows intermolecular interactions indicated by green dotted lines, anion···π (3.371 Å) and π···π (3.315 Å) [for (a) anion···π (3.835 Å), for (b) anion···π (3.712 Å)]. (d) Schematic representation of the portion shown inside the dashed box in (c) exemplifying probable anion···π mediated charge transfer hopping (indicated by a green arrow) of the localized charges of dca^–^ through RPh_3_P^+^ moieties.

In this work, we use a unique organic A cation which has a different electronic state of the central element than the previously detailed family to investigate this structure–property correlation. We modify the type of organic A cation and study (RPh_3_P)[Mn(dca)_3_] (where R = Me, Et, Ph) structures through the introduction of methyltriphenylphosphonium (MePh_3_P = (CH_3_)(C_6_H_5_)_3_P^+^), ethyltriphenylphosphonium (EtPh_3_P = (CH_3_CH_2_)(C_6_H_5_)_3_P^+^) and tetraphenylphosphonium (Ph_4_P = (C_6_H_5_)_4_P^+^) species (Figure 1a–c).^30,31^ Previous tetraalkylammonium-based cations contained a quaternary nitrogen with 2p valence shell.^21^ Here, we use cations including a quaternary phosphorus (vacant 3d orbital) linked to aromatic phenyl moieties having delocalized π electron clouds. This allows the investigation of potential charge transport such as charge/electronic hopping occurring between the dicyanamide X linkers (having free localized charges) via dπ···pπ coupled RPh_*x*_P moieties as indicated in Figure 1d. We study the melting and glass transition features and explore multiple physical properties of the melt-quenched glasses formed from these materials having dπ···pπ coupled phenylphosphonium A cations.

## Results and Discussion

### Structural Analysis of Crystalline Materials

The synthesis of (MePh_3_P)[Mn(dca)_3_],^30^ (EtPh_3_P)[Mn(dca)_3_],^31^ and (Ph_4_P)[Mn(dca)_3_]^30,32^ materials was carried out as previously reported and their phase purities were checked by Pawley refinement of powder X-ray diffraction (PXRD) data collected on bulk samples (Figure S1).

In the past decade, [Mn(dca)_3_]^−^ networks have exhibited various structural building units such as polymeric one-dimensional (1D) chains,^32,33^ two-dimensional (2D) layers,^34,35^ and three-dimensional (3D) networks.^31,36^ Here, with the tetraphenylphosphonium cation (Ph_4_P^+^), the [Mn(dca)_3_]^−^ anion forms a 2D square lattice.^30^ However, minor changes in the cationic molecular template have been shown to have dramatic effects on the topology of the [Mn(dca)_3_]^−^ network. Substitution of one phenyl ring with a smaller methyl/ethyl group (RPh_3_P^+^; R = Me, Et) yields a 3D distorted triple rutile-type (MePh_3_P)[Mn(dca)_3_] and (EtPh_3_P)[Mn(dca)_3_] structures. These have doubly μ_1,5_-dca bridged dimerized manganese units joined together through single μ_1,5_-dca linkages.^30,31^ Introduction of smaller cations actually disrupt the subtle cation–cation and cation–anion interactions, and reduces the packing efficiency of the cation layer with structural flexibility of the [Mn(dca)_3_]^−^ network.^37^ The A cation thus exhibits a templating function and lies in pairs within cavities in the anionic network, rather than in the discrete layers as seen in the (Ph_4_P)[Mn(dca)_3_] framework. The cations lie between the sheets and display cation–cation interactions of the π–π and pseudo-6-fold phenyl embrace types.^31,38^

### Thermal Analysis

**Figure 2:**
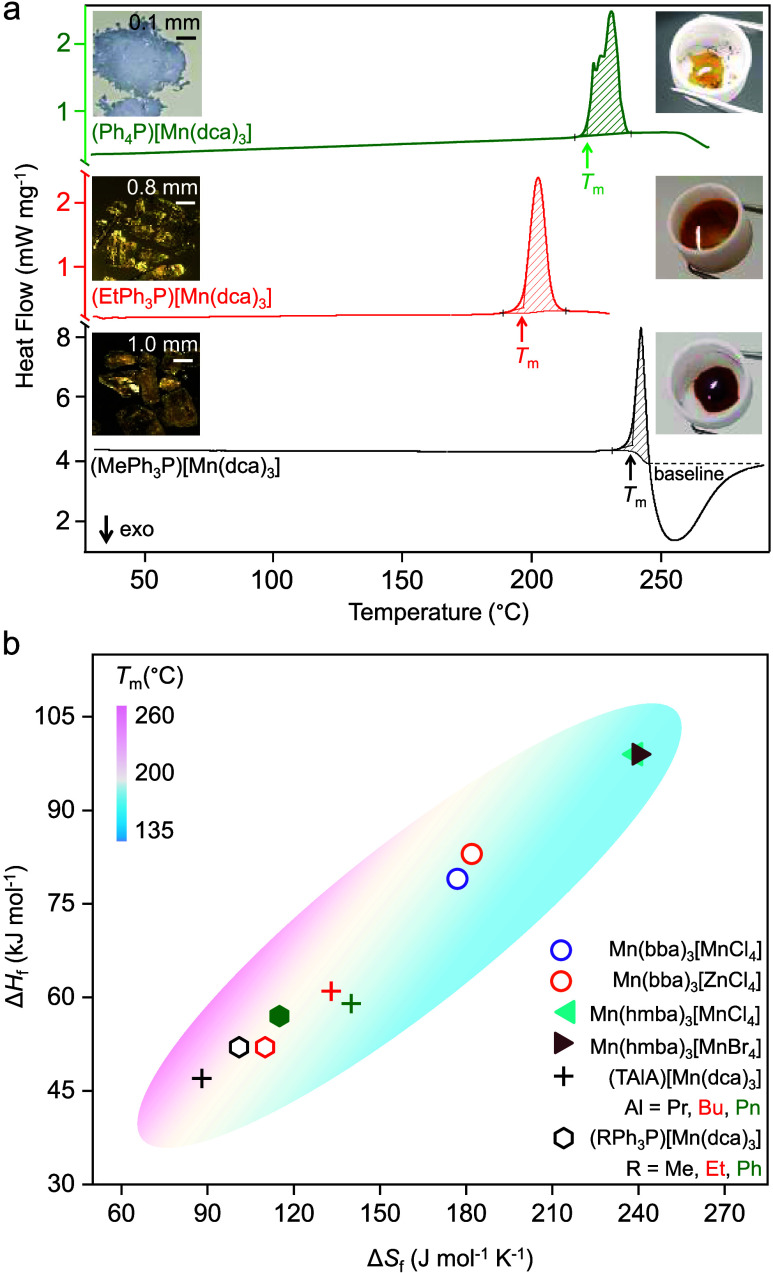
(a) Change in heat flow with an increase in temperature for (RPh_3_P)[Mn(dca)_3_] crystalline samples. *T*_m_’s were determined from change in slope at onset. Δ*H*_f_ for the crystalline to liquid transition were extracted from the shaded sigmoidal areas. For (MePh_3_P)[Mn(dca)_3_], Δ*H*_f_ was determined after subtracting a baseline (black dash). Likely, the different shape of the heat profile postmelting is associated with partial degradation. The value without baseline is shown in Table S1. The inset shows the optical images of the crystalline solid (left) and molten liquid (right) taken instantly after opening the heating furnace near the *T*_m-offset_. (b) Comparison of Δ*H*_f_ and Δ*S*_f_ for the present materials with our previously reported (TAlA)[Mn(dca)_3_]^21^ and various other 2D and 3D bis(acetamide)-based MOFs.^41^ “Hexagon” and “plus” symbols were chosen to specifically differentiate between phenyl and tetraalkyl cation-based systems. Filled symbols represent 2D materials.

Thermogravimetric analysis (TGA) of (RPh_3_P)[Mn(dca)_3_] crystalline samples exhibit temperatures of decomposition (*T*_d_) at 288, 285, and 308 °C for R = Me, Et, Ph analogues, respectively (Figure S2). Differential scanning calorimetry (DSC) was then performed to identify possible phase changes prior to *T*_d_ (Figure 2a). All of the compounds show a melting endotherm below *T*_d_ upon heating in the first upscan, and it is noted that their values of *T*_d_ and *T*_m_ appear close to those with alkylammonium series due to the same M-dca network connectivity in these hybrid structures (Table 1). Interestingly, the melting endotherm of the 2D (Ph_4_P)[Mn(dca)_3_] structure, with a peak value of 231 °C, was associated with two minor peaks at 222 and 224 °C.^39^ Potentially, this points at a sluggish structural transformation kinetics, as previously observed for 2D hybrid perovskites in conventional DSC techniques,^4^ 2D CPs,^40^ and semicrystalline organic polymers.^39^ Similar to previous results for 3D alkylammonium series,^21^ a decreasing trend in *T*_m_ values with increasing size of the R groups in the A site cation is observed in the 3D compounds, i.e., (EtPh_3_P)[Mn(dca)_3_] (*T*_m_ = 196 °C) < (MePh_3_P)[Mn(dca)_3_] (*T*_m_ = 237 °C). As enthalpy change (Δ*H*_f_) at melting is correlated directly with M–N coordination bond strength, their variation (52–57 kJ mol^–1^) appears insignificant due to the same M–N (Mn–N) connectivity in all three compounds. The slightly higher Δ*H*_f_ for (Ph_4_P)[Mn(dca)_3_] (57 kJ mol^–1^, Figure 2b) is consistent with its high extent of subtle cation–cation and cation–anion interactions.^40^ Despite having a similar enthalpic variation, a nearly 40 °C increase in *T*_m_ for 3D (MePh_3_P)[Mn(dca)_3_] with respect to that of 3D (EtPh_3_P)[Mn(dca)_3_] is fully compensated for by a decrease in its change in entropy (Δ*S*_f_, 110–101 J mol^–1^ K^–1^), as seen in the case of network-forming bis(acetamide) structures.^41^ Weight losses of less than 0.2% were observed at *T*_m-offset_ in all the cases.

### Glass Formation

As in our previous reports on (TAlA)[M(dca)_3_] samples,^17^ opaque, glass-like pieces were observed after cooling the (RPh_3_P)[Mn(dca)_3_] melts from their offset of melting temperatures (Figure S3) and found to be amorphous by X-ray diffraction (Figure S4). For (MePh_3_P)[Mn(dca)_3_] and (EtPh_3_P)[Mn(dca)_3_], ground crystals were heated 30 °C above their melting maximum, and then cooled to −50 °C at a heating–cooling rate of 10 °C min^–1^, to form glass samples. In the case of (Ph_4_P)[Mn(dca)_3_], an exothermic recrystallization was seen while cooling the melt at the same rate of 10 °C min^–1^ (Figure S5a), so we applied a slower cooling rate (3 °C min^–1^) to prevent the exothermic event and obtain the complete glass phase (Figure S5b).^21^ This is the same counterintuitive approach applied previously with similar dca-based species, which were prone to recrystallization during cooling and ascribed to partial decomposition being necessary in order for glass formation to occur. The glasses formed, in keeping with existing nomenclature on hybrid glasses, are thus termed *a*_g_(RPh_3_P)[Mn(dca)_3_] (*a*_g_: melt-quenched glass).

**Figure 3:**
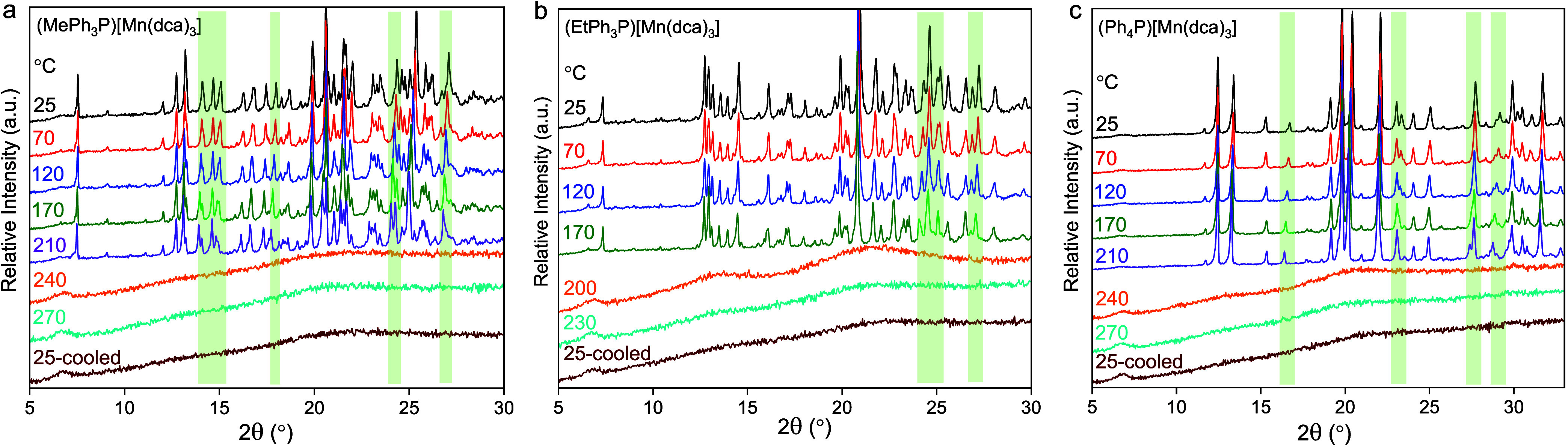
(a–c) Variable-temperature PXRD patterns of crystalline (RPh_3_P)[Mn(dca)_3_] samples. During the heating scans, a rate of 10 (±1) °C min^–1^ was applied. To comply with the DSC measurements, the maximum instrumental cooling was applied for (a) and (b) which provides a rate of 9 (±1) °C min^–1^. For (c), we have opted for a cooling rate of 5 °C min^–1^ which provides 3 (±1) °C min^–1^. The absolute temperatures during each 2θ scan were found to be varied by ±1 °C. Green transparent regions indicate the change of Bragg peak shape and position with an increase in temperature. Small hump at 2θ = 6.7° in every scan appeared from the instrument background.

The crystal to amorphous phase transition upon melt-quenching was monitored by using the variable-temperature powder X-ray diffraction (VT-PXRD) experiments (Figure 3). Isothermal XRD experiments were conducted using the “constant up-down measurement temperature loop” mode (Figure S6). To avoid longer exposure of heat to the samples during each elevated temperature scan, a limited window of 2θ was chosen up to which peaks with only high-intensity exist (Figure S1) i.e., 2θ = 5–30° for (MePh_3_P)[Mn(dca)_3_] and (EtPh_3_P)[Mn(dca)_3_] and 5–33° for (Ph_4_P)[Mn(dca)_3_]. The compounds retain crystallinity before their melting onset. PXRD patterns were taken at their corresponding melting peak temperatures, i.e., at 240 °C for (MePh_3_P)[Mn(dca)_3_], (Ph_4_P)[Mn(dca)_3_] and 200 °C for (EtPh_3_P)[Mn(dca)_3_], reveal no Bragg peaks and imply liquefication. To comply with the DSC measurements, we heated the liquids 30 °C above their melting maximum before cooling back to room temperature. The PXRD patterns of quenched phases at room temperature follow their high-temperature liquid phase and reveal complete amorphization. The optical images of the samples before and after melt-quenching are shown in Figure S7.

**Figure 4:**
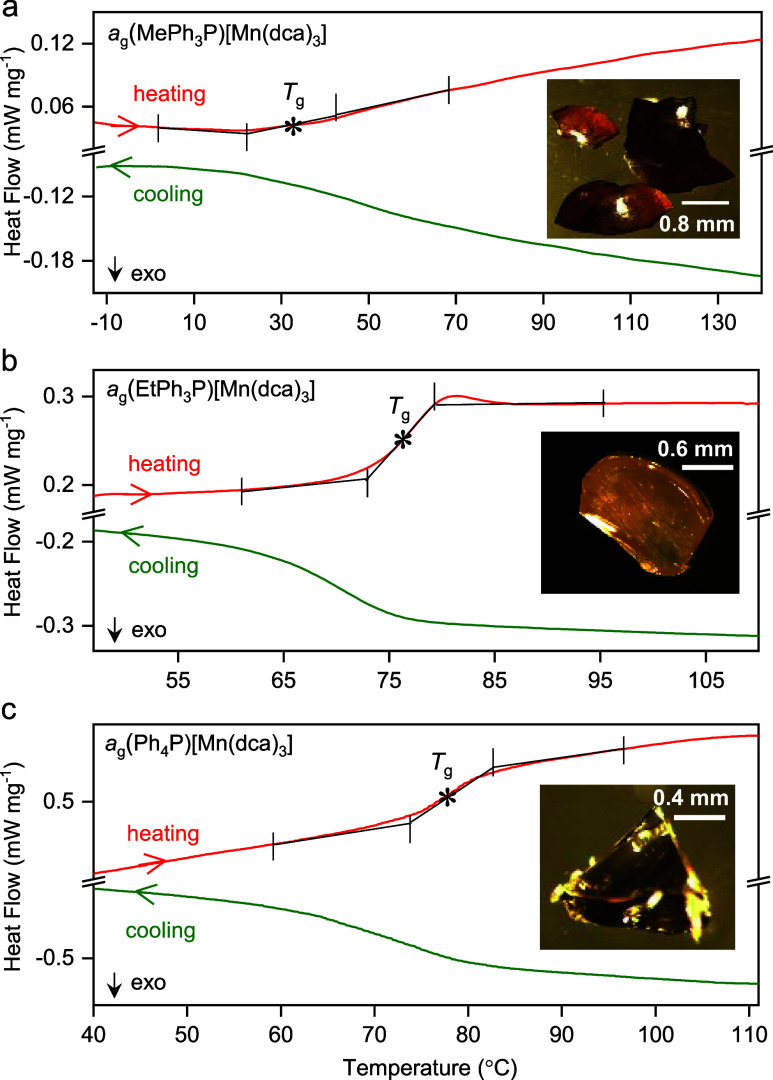
Change in heat flow as a function of temperature for *a*_g_(RPh_3_P)[Mn(dca)_3_]. The green lines show cooling of the molten liquids down to below room temperature, and then reheating of the quenched glasses is shown by red lines. The onset of glass transition (*T*_g_) was evaluated from reheating curves and marked with an asterisk.^42^ Complete cycles of heating–cooling–reheating runs are shown in the Supporting Information (Figures S8–S10). A rate of 10 °C min^–1^ for cooling–reheating was applied for *a*_g_(MePh_3_P)[Mn(dca)_3_] and *a*_g_(EtPh_3_P)[Mn(dca)_3_], and 5 °C min^–1^ for *a*_g_(Ph_4_P)[Mn(dca)_3_], respectively. For *a*_g_(MePh_3_P)[Mn(dca)_3_], the different shapes of the heat profile at the glass transition are associated with the partial degradation explained earlier. Insets show the optical images of each of the glasses.

Cooling of the molten liquids and subsequent reheating of the quenched glass yielded smooth changes in the heat flow of the glass transitions (*T*_g_) at 33, 75, and 77 °C for *a*_g_(MePh_3_P)[Mn(dca)_3_], *a*_g_(EtPh_3_P)[Mn(dca)_3_] and *a*_g_(Ph_4_P)[Mn(dca)_3_], respectively (Figures 4, S8–S10 and Table 1). Unlike alkylammonium-based glasses which behaved mostly like dca-based ionic liquids (*e.g., T*_g_ = −26 °C for *a*_g_(TPnA)[Co(dca)_3_],^21^ −67 °C for [N_8444_][dca]^43^), the higher *T*_g_ above room temperature for the present glasses show a higher stability and also indicates the strong cation dependency of the glass transition temperatures. This is also supported by its high values of *T*_g_/*T*_m_ from the empirical “*T*_g_/*T*_m_ ∼ 2/3” values, such as 0.74 for *a*_g_(EtPh_3_P)[Mn(dca)_3_] and 0.71 for *a*_g_(Ph_4_P)[Mn(dca)_3_]. Interestingly, despite the two dissimilar structural topologies of the *a*_g_(EtPh_3_P)[Mn(dca)_3_] and *a*_g_(Ph_4_P)[Mn(dca)_3_] precursors, their *T*_g_/*T*_m_ values appear to be close. The low *T*_g_/*T*_m_ ∼ 0.60 for *a*_g_(MePh_3_P)[Mn(dca)_3_] indicates its low glass-forming ability compared to other two analogues, *a*_g_(EtPh_3_P)[Mn(dca)_3_] and *a*_g_(Ph_4_P)[Mn(dca)_3_].^44^ Overall, a close proximity of *T*_g_/*T*_m_ was observed between all dicyanamide-based hybrid organic–inorganic structures (Figure S11).

**Figure 5:**
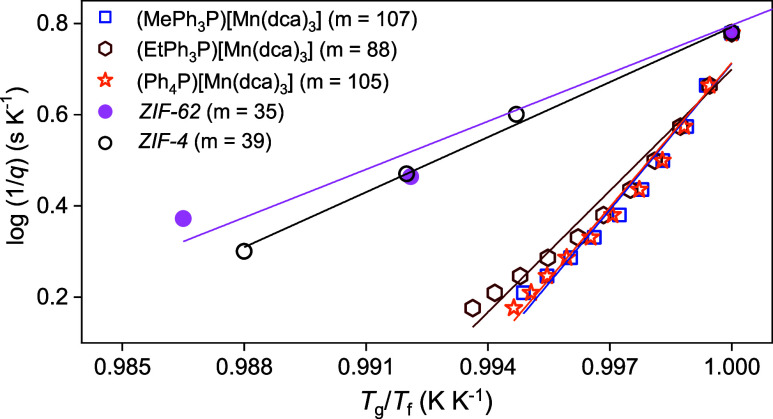
Calorimetric fragility indices (*m*) of (RPh_3_P)[Mn(dca)_3_] are shown with various ZIF-based systems. The values were evaluated from the dependence of the fictive temperature (*T*_f_) on the heating rate (*q*). The samples were heated from 10 to 40 °C min^–1^ with 3 °C intervals. The cycles of cooling–reheating of the molten liquid are shown in Figures S12–S14.

To study the transition dynamics of the molten liquid as it approaches the glassy phase, we have evaluated the kinetic fragility index, *m*, using the DSC method (Figures 5 and S12–S14). Compared to ZIF-based strong liquids, which exhibit lower fragilities (e.g., ZIF-62, *m* = 39)^45^ and form brittle glasses like silica (*m* = 20), here, the higher values of *m* (88–107) indicate the fragile nature of hybrid organic–inorganic liquids and so can be categorized as ductile glasses (e.g., toluene, *m* = 105). The slightly low value of *m* for *a*_g_(MePh_3_P)[Mn(dca)_3_] is in line with its low glass-forming ability (*T*_g_/*T*_m_) compared to the other two analogues.

A minimal 0.6–1.0% gravimetric mass loss was detected upon formation of glasses via the above quenching techniques (Figure S15). Elemental analyses (C, N, H, P) of both crystalline and glassy phases were performed to evaluate any change in composition during melt-quenching (Table S2). Around 6.0% mass loss was seen for C and N in *a*_g_(MePh_3_P)[Mn(dca)_3_]. 2.2% amount of C was lost in *a*_g_(EtPh_3_P)[Mn(dca)_3_] and 2.0% in *a*_g_(Ph_4_P)[Mn(dca)_3_]. No significant change is observed in any sample for H or P. This is also reflected in their FT-IR spectra, where two (weak) new bands at 1629–1634 and 802–806 cm^–1^ appear, indicating high-temperature deformation of a portion of dca ligand (vibration of δ_C–N–C_) during melt-quenching as reported previously (Figure S16).^17,46−48^

### Structural Changes upon Glass Formation

#### Nuclear Magnetic Resonance and High-Resolution Mass Spectrometry

**Figure 6:**
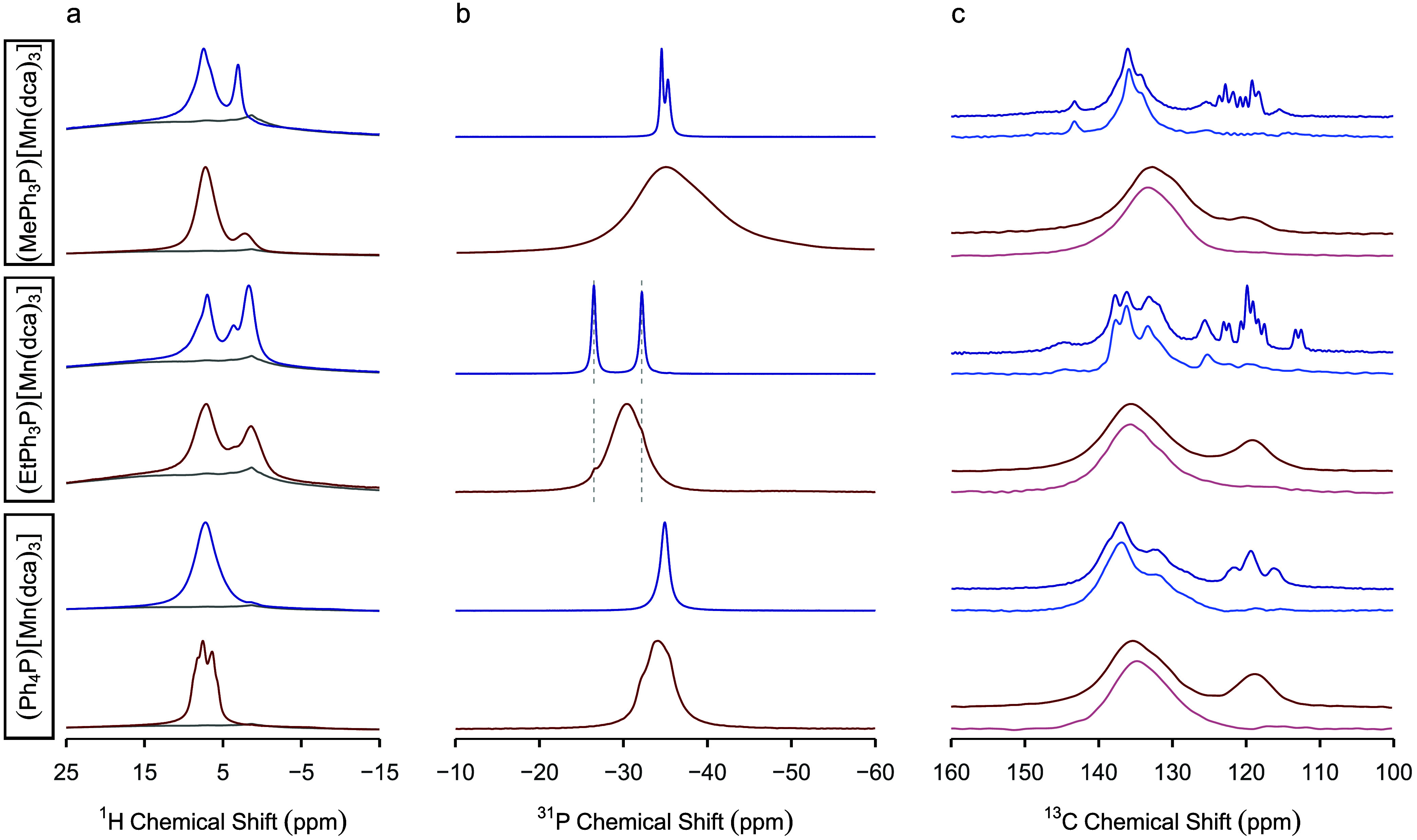
(a) ^1^H, (b) ^31^P, and (c) ^13^C double adiabatic echo MAS NMR spectra of (blue) crystalline (RPh_3_P)[Mn(dca)_3_] and (red) *a*_g_(RPh_3_P)[Mn(dca)_3_]. The ^1^H MAS NMR spectra were recorded at 18.8 T. The gray spectra below the ^1^H spectra indicate the probe background. The ^31^P and ^13^C MAS NMR spectra were recorded at 9.4 T. The ^13^C MAS NMR spectra with full spectral width are shown in Figure S18. The dashed lines in (b) correspond to the positions of the two ^31^P signals in the spectrum of crystalline (EtPh_3_P)[Mn(dca)_3_]. One-dimensional ^1^H ^13^C TEDOR MAS NMR spectra are shown in a lighter shade of blue/red below the corresponding ^13^C double adiabatic echo MAS NMR spectra.

^1^H, ^31^P, and ^13^C magic angle spinning (MAS) nuclear magnetic resonance (NMR) spectra of (RPh_3_P)[Mn(dca)_3_] before and after melt-quenching are shown in Figures 6, S17, and S18. They were recorded under optimized conditions for paramagnetic solids^49,50^ at very fast MAS rates (60 kHz) due to the presence of the Mn(II) paramagnets. A full description of all NMR data for crystalline (RPh_3_P)[Mn(dca)_3_] is given in the Supporting Information but is compared to those for *a*_g_(RPh_3_P)[Mn(dca)_3_] here.

The ^1^H MAS NMR spectra of crystalline (RPh_3_P)[Mn(dca)_3_] are broadly comparable to those of the corresponding melt-quenched glasses. For example, the ^1^H MAS NMR spectra of both crystalline and glassy (EtPh_3_P)[Mn(dca)_3_] display one signal in the aromatic region which is assigned to the protons of the phenyl rings and two signals in the aliphatic region that correspond to the –C*H*_2_ and –C*H*_3_ units of the ethyl group. Nevertheless, the signals in the spectra of *a*_g_(RPh_3_P)[Mn(dca)_3_] are generally broader compared to those observed for crystalline (RPh_3_P)[Mn(dca)_3_]. This inhomogeneous broadening arises from the distribution of chemical shifts typical of disordered solids^51^ and supports the amorphous state of the melt-quenched glasses. The ^1^H MAS spectrum of *a*_g_(Ph_4_P)[Mn(dca)_3_] unexpectedly exhibits enhanced spectral resolution compared to the spectrum of crystalline (Ph_4_P)[Mn(dca)_3_] which might be due to different dynamics. Compelling evidence of the presence of the RPh_3_P^+^ cations in *a*_g_(RPh_3_P)[Mn(dca)_3_] is provided by the ^31^P MAS NMR spectra that display one broad peak at a chemical shift approximately equal to the average position of the resonances observed for the crystalline materials (see the Supporting Information for a full discussion of the MAS NMR spectra of the crystalline phases), thus confirming amorphization upon melt-quenching. Close inspection of the ^31^P MAS NMR spectrum of *a*_g_(EtPh_3_P)[Mn(dca)_3_] reveals the presence of two tiny residual signals corresponding to crystalline (EtPh_3_P)[Mn(dca)_3_] and indicates nearly complete amorphization (confirmed by the X-ray total scattering data of the sample, see the X-ray Pair Distribution Function Analysis section, Figure S29). As for the ^13^C MAS NMR spectra of *a*_g_(RPh_3_P)[Mn(dca)_3_], two main broad signals can be observed in the 150–100 ppm region that coincide to a great extent with the envelope of the signals in the spectra of the corresponding crystalline (RPh_3_P)[Mn(dca)_3_] samples (Figure S18). The absence of the broad signal at ∼118 ppm in the ^1^H ^13^C Transfer Echo Double Resonance (TEDOR) MAS NMR spectra of *a*_g_(RPh_3_P)[Mn(dca)_3_] (Figure 6c) indicates that this resonance corresponds to the quaternary carbons in the dca^–^ and RPh_3_P^+^ units, similar to what is observed for the crystalline (RPh_3_P)[Mn(dca)_3_] materials. Two broad and partially overlapping signals assigned to the –*C*H_2_ and –*C*H_3_ units of the ethyl group are present in the aliphatic region of the ^13^C MAS NMR spectrum of *a*_g_(EtPh_3_P)[Mn(dca)_3_], while the poor signal-to-noise ratio makes it challenging to observe the –*C*H_3_ signal in the spectrum of *a*_g_(MePh_3_P)[Mn(dca)_3_] (Figure S18).

Overall, the comparison of the MAS NMR spectra of crystalline and glassy (RPh_3_P)[Mn(dca)_3_] highlights differences in line broadening that are indicative of the formation of disordered solids and similarities in chemical shift that demonstrate the retention of the overall composition upon amorphization.

High-resolution mass spectrometry (HRMS) as well as ^1^H, ^31^P, and ^13^C liquid-phase NMR data of the crystalline and glassy (RPh_3_P)[Mn(dca)_3_] materials digested under acidic conditions confirm the identity of the building blocks of the hybrid organic–inorganic ABX_3_ structures. HRMS data with ionization in positive or negative mode (Figures S19–S24) demonstrate the presence of the RPh_3_P^+^ cations or the dca^–^ anions, respectively, in all of the digested samples. For example, the HRMS data obtained for the crystalline and glassy (Ph_4_P)[Mn(dca)_3_] digested samples present intense peaks at *m*/*z* values of 66.0101 and 339.1301 which correspond to the dca^–^ anion and Ph_4_P^+^ cation, respectively. The ^1^H liquid-phase NMR spectra of (RPh_3_P)[Mn(dca)_3_] and *a*_g_(RPh_3_P)[Mn(dca)_3_] (R = Me, Et) (Figure S25) show typical signals for aliphatic moieties in the 4–1 ppm region. For example, signals corresponding to the –C*H*_2_ (3.4 ppm) and –C*H*_3_ (1.0 ppm) units of the ethyl group are observed for (EtPh_3_P)[Mn(dca)_3_] and *a*_g_(EtPh_3_P)[Mn(dca)_3_]. In all of the ^1^H NMR spectra, either one signal or many overlapping signals corresponding to the protons in the phenyl rings are observed at ∼7.5 ppm. An additional signal above 8 ppm is observed in most ^1^H NMR spectra and is tentatively assigned to an H_2_O-protonated dca^–^ ligand which exists as an aminonitrile-carbodiimide tautomer in acidic conditions.^17^ Importantly, one ^31^P signal assigned to the RPh_3_P^+^ units is observed in all of the ^31^P NMR spectra (Figure S26). ^13^C NMR signals corresponding to the –*C*H carbons of the phenyl rings are clearly present in all the spectra (Figure S27), while poor signal-to-noise ratio challenges the detection of both dca^–^ units and aliphatic carbons (Figure S28), the presence of which is already established from the HRMS and ^1^H spectra.

### X-ray Pair Distribution Function Analysis

**Figure 7:**
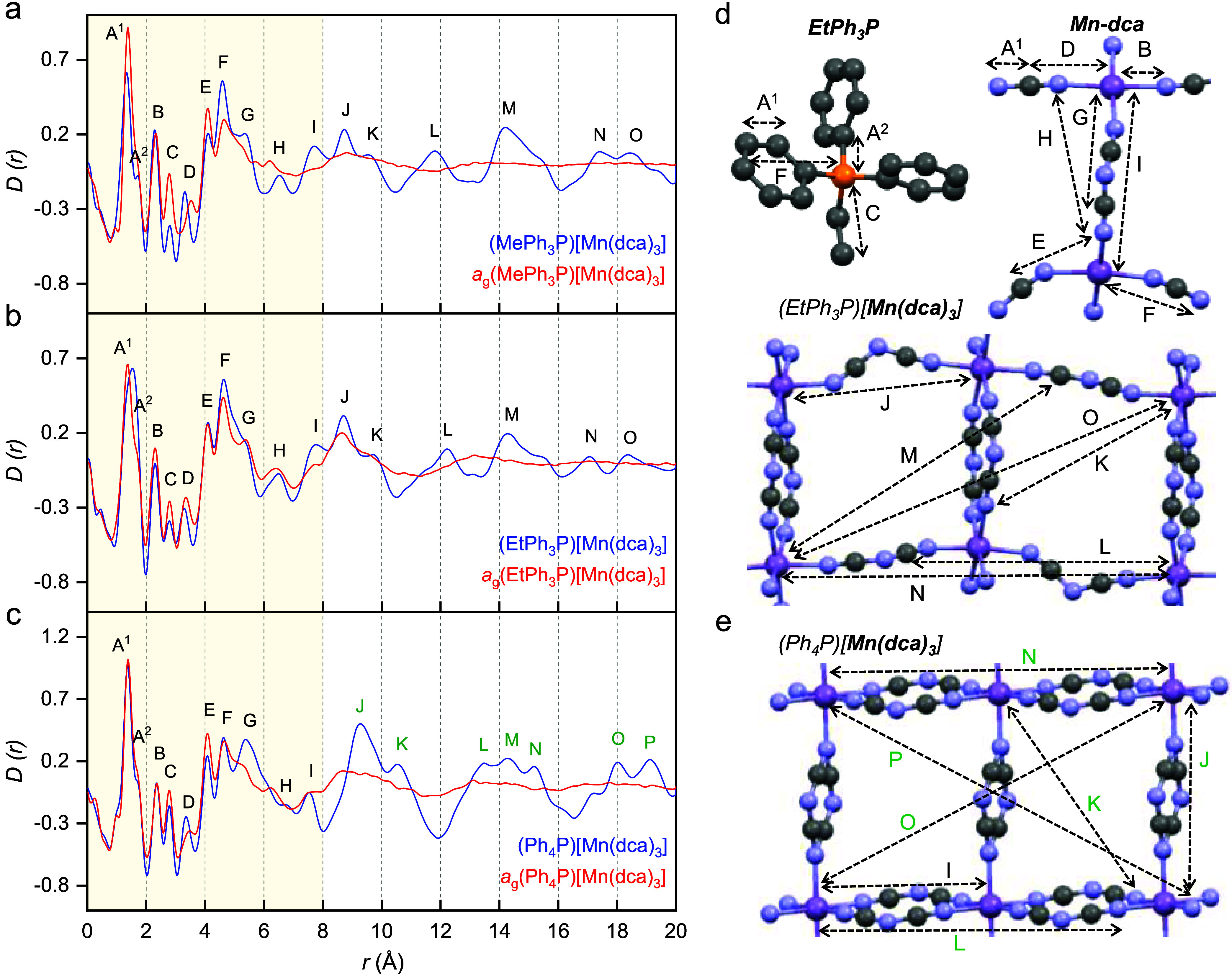
(a–c) Pair distribution functions for (RPh_3_P)[Mn(dca)_3_] and *a*_g_(RPh_3_P)[Mn(dca)_3_] at room temperature. (d) As (MePh_3_P)[Mn(dca)_3_] and (EtPh_3_P)[Mn(dca)_3_] exhibit similar network connectivity, we have used the A cation and extended Mn-dca network of the (EtPh_3_P)[Mn(dca)_3_] published CIF at 298 K^52^ to identify the peak positions (purple: Mn; orange: P; gray: C; blue: N); H atoms are omitted for clarity. (e) Extended Mn-dca network of (Ph_4_P)[Mn(dca)_3_] at 298 K.^52^ Reproduced from ref (52). Copyright [2005] American Chemical Society.

To provide atomic-level insight into the changes in bonding upon melt-quenching into the glassy state, room-temperature X-ray total scattering experiments were performed on the crystalline and glass (RPh_3_P)[Mn(dca)_3_] samples. The structure factors, *F*(*Q*), of the crystalline materials show Bragg peaks arising from long-range order, while, the data of corresponding glasses, *a*_g_(RPh_3_P)[Mn(dca)_3_], show smooth broad humps indicating the loss of long-range crystalline order upon melt-quenching (Figures S29 and S30a).^21^ The *F*(*Q*) of (MePh_3_P)[Mn(dca)_3_] and (EtPh_3_P)[Mn(dca)_3_] (Figure S29a,b) look similar to each other due to their similar structural topology but differ from (Ph_4_P)[Mn(dca)_3_], which possesses a completely different framework (Figure S29c). The pair distribution functions (PDFs), *D*(*r*), were extracted after appropriate data corrections using experimental pycnometric densities (Table S3). These show a similar trend (Figure 7a–c). To aid the assignment of the peaks in the PDFs, the published structures for the crystals were refined using the PDF data, and weighted partial pair distributions, *g*_*ij*_(*r*), were calculated (Figures S31–S33).

Owing to the similar network connectivity of (MePh_3_P)[Mn(dca)_3_] and (EtPh_3_P)[Mn(dca)_3_] compounds, their PDF peak widths and positions appear similar up to 20 Å (Figure 7a,b). Despite (Ph_4_P)[Mn(dca)_3_] having a different structural symmetry, its short-range peak positions (up to 8.0 Å, indicated by the region inside the yellow shade) match (Figure 7c) those of the above structures well, as these are related to the correlations present between the atoms in a single Mn-dca linkage and those between the atoms within RPh_3_P cations (A⃡–G⃡, Figure 7d). Correlations at high *r* (>8 Å) are dominated by those between atoms in adjacent Mn-dca pairs (K⃡–P⃡), and as such are more heavily influenced by the large size of the R (phenyl) group in (Ph_4_P)[Mn(dca)_3_] (symbols marked by green in Figure 7e). The correlation at *r* = 1.3 Å, contains contributions predominately from C–C and C≡N atom pairs (labeled A⃡^1^). The small side peak at 1.7 Å corresponds to P–C correlations (A⃡^2^). The correlation at *r* = 2.3 Å is mainly ascribed to the Mn–N pair (B⃡). The peaks at 2.8 and 3.4 Å are ascribed to the P···C (C⃡, via the P–C–C linkage) and Mn···C (D⃡, via the Mn–N–C linkage) correlations. Three peaks at 4.1, 4.6, and 5.4 Å represent N···C (E⃡), Mn···N (F⃡), and Mn···C (G⃡) correlations, respectively. The two, short (I⃡) and long (J⃡) Mn···Mn correlations are at *r* = 7.8 and 8.8 Å for (MePh_3_P)[Mn(dca)_3_] and (EtPh_3_P)[Mn(dca)_3_], and at 7.6 and 9.3 Å for (Ph_4_P)[Mn(dca)_3_], respectively.

For all three *a*_g_(RPh_3_P)[Mn(dca)_3_] glasses, their *F*(*Q*) and *D*(*r*) look similar to each other, showing that they have similar atom–atom correlations in the glassy phase after melt-quenching (Figure S30). Unlike our previous results for the alkylammonium series of *a*_g_(TAlA)[M(dca)_3_] glasses,^21^ the *D*(*r*) of *a*_g_(RPh_3_P)[Mn(dca)_3_] glasses show a strong resemblance to their crystalline precursors up to 8.0 Å. Correlations between 8.0 and 12.0 Å were, however, reduced in intensity and substantially broadened and then were near negligible above 12.0 Å. This is consistent with the mechanism of melting involving the breakage of Mn–N bonds, and the associated movement of the RPh_3_P cations out of the A site cavity.

### Magnetic Study

Temperature-dependent DC magnetic susceptibility was investigated over the temperature range from 10 to 298 K. Variation of the effective magnetic moment, μ_effective_ (calculated from χ_M_: molar susceptibility, μ(B.M.) = 2.83√χ_M_*T*), is plotted as a function of *T* (Figure S34a).^53^ The room-temperature magnetic moment (μ_RT_) for all crystalline phases is in line with the number of unpaired spins present in high spin Mn(II) (∼5.92 Bohr Magneton at 298 K).^30,31^ The decreasing trend at lower temperatures implies the effect of single-ion zero-field splitting and a weak antiferromagnetic coupling between the Mn(II) (^6^A_1g_) metal centers.^28^ Interestingly, due to a severe temperature-independent paramagnetic (TIP) contribution in the *a*_g_(RPh_3_P)[Mn(dca)_3_] glasses, the μ_RT_ values become high and unevaluable (Figure S34b).

### Mechanical Property of *a*_g_(RPh_3_P)[Mn(dca)_3_]

The mechanical strength of melt-quenched glass samples was probed by the nanoindentation method. The load versus displacement behavior (Figure S35) revealed the Hardness (*H*) of the glasses which basically reflects the resistance of a material to plastic deformation. Values of *H* range between 0.43 and 0.47 (±0.02) GPa which are comparable to various MOF glasses e.g., 0.7 for *a*_g_ZIF-62, 0.9 for *a*_g_ZIF-4.^45^

### Electrical and Thermal Conductivity Study of *a*_g_(RPh_3_P)[Mn(dca)_3_]

**Figure 8:**
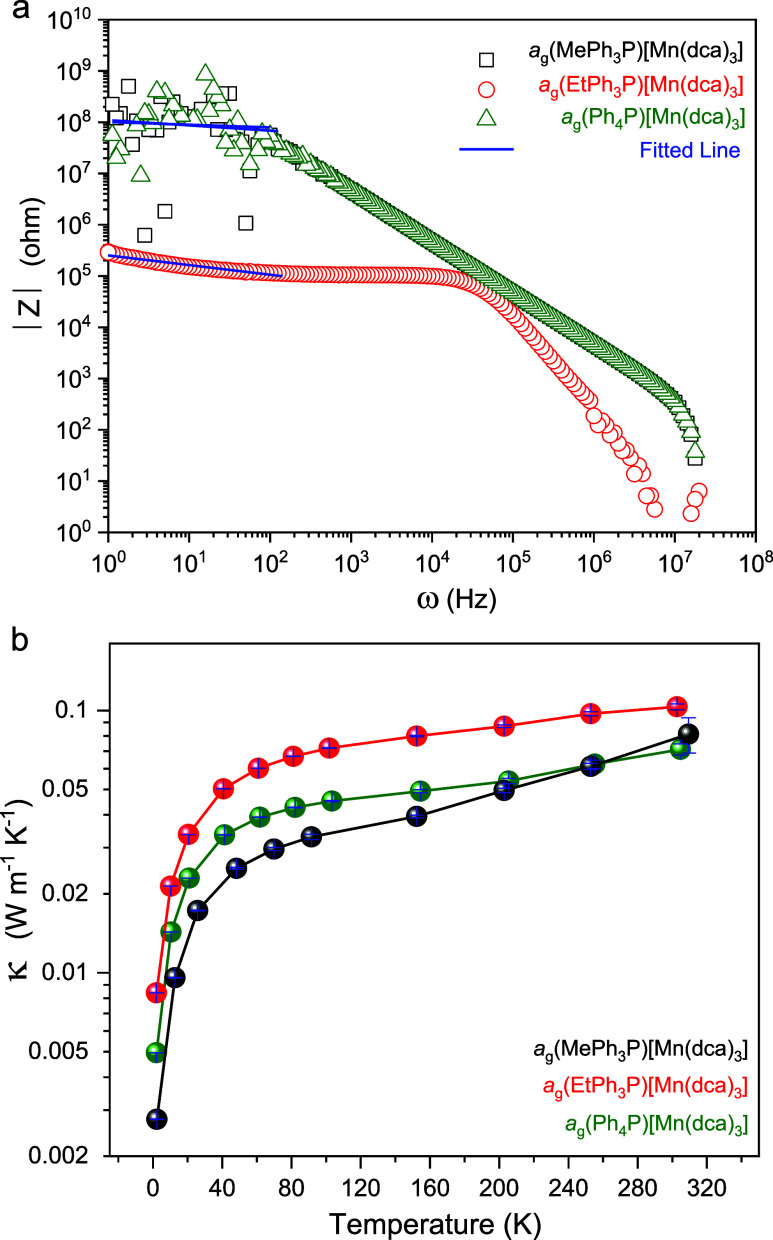
(a) Room-temperature AC impedance spectroscopy data of *a*_g_(RPh_3_P)[Mn(dca)_3_]. The DC resistance values were evaluated from the cutoff point of the fitted theoretical lines. (b) Logarithmic variation of thermal conductivity (κ) as a function of temperature from 10 to 298 K for *a*_g_(RPh_3_P)[Mn(dca)_3_].

Frequency-dependent AC electrical conductivity measurements were carried out to probe the charge transport behavior of these newly designed phenylphosphonium-based *a*_g_(RPh_3_P)[Mn(dca)_3_] glasses. Values of DC resistance were evaluated after fitting the variation of |*Z*| versus ω (Figure 8a). Room-temperature DC conductivities (σ_RT_) of 0.5 (±0.2) and 0.4 (±0.2) (10^–6^ S m^–1^) for *a*_g_(MePh_3_P)[Mn(dca)_3_] and *a*_g_(Ph_4_P)[Mn(dca)_3_], respectively, characterized these as weakly conducting materials. *a*_g_(EtPh_3_P)[Mn(dca)_3_] however appears to be moderately conductive with a value of 0.8 (±0.05) (10^–4^ S m^–1^). A high degree of charge transport was expected, e.g., by charge hopping between the dicyanamide and dπ···pπ coupled RPh_3_P moieties (as demonstrated in Figure 1d). However, instead, the weak electronic inter- or intrachain coupling between the X linkers dominates, making these moderately conducting, similar to the alkylammonium-containing compounds.^17,54^

The thermal conductivity (κ) is of great importance given the potential application of hybrid ABX_3_ glasses as thermoelectric materials for waste heat power generation and cooling of electronics. The κ values of the *a*_g_(RPh_3_P)[Mn(dca)_3_] samples were probed, finding absolute values at room temperature (κ_RT_) of 0.07–0.09 W m^–1^ K^–1^ (Figure 8b). The close values suggest that the thermally insulating organic component (similar *r*_A_eff__) predominates over the thermally conductive metallic components. These values are relatively low compared to materials reported in the literature, e.g., Bi_4_O_4_SeCl_2_ (∼0.1 W m^–1^ K^–1^), MOF-5 (0.32 W m^–1^ K^–1^), organic–inorganic lead halide hybrid perovskites (∼0.40 W m^–1^ K^–1^) and doped silicate or borosilicate glasses (∼1 W m^–1^ K^–1^).^3,55−58^ By the incorporation of the phenylphosphonium derivatives in the network, the κ value has been significantly decreased compared to the alkylammonium derivative glasses and makes them promising candidates as thermal insulator materials or as functional fillers, like Perlite (0.07 W m^–1^ K^–1^).

## Conclusions

The results reported in this work demonstrate the effect of the RPh_3_P^+^ (where R = Me, Et, Ph) A cation on the melting and vitrification of ABX_3_-type hybrid organic–inorganic materials. We have established a basis of comparison for three phenylphosphonium-based (RPh_3_P)[Mn(dca)_3_] structures with alkylammonium-based (TAlA)[Mn(dca)_3_] materials and conducted a thorough study of their thermal properties derived from their diverse chemical structure. An increase in the size of R groups reduces the dimensionality of the material and was observed to influence the melting feature, melting temperature (*T*_m_), and corresponding changes in enthalpy (Δ*H*_f_) and entropy (Δ*S*_f_). An increase in the size of the R group in the A site cation was found to decrease the *T*_m_ in the 3D materials. Also, despite having comparable enthalpy changes (Δ*H*_f_) for 3D (MePh_3_P)[Mn(dca)_3_] and (EtPh_3_P)[Mn(dca)_3_] materials, the former was seen to exhibit a much higher *T*_m_. This is compensated for by a decrease in the entropy changes (Δ*S*_f_). Large-scale production of glasses via melt-quenching technique was directly established using in situ variable-temperature PXRD experiment. Solid-state NMR and PDF analysis displayed the intactness of the bonding between the metal centers and bridging dicyanamide ligands in the glassy phases. The newly designed glasses show a *T*_g_ above room temperature and possess a high glass-forming ability compared to alkylammonium-based glasses and various other metal-halide perovskites (MHPs). In addition to this, the glasses designed here were mechanically firm, durable, and similar to ZIFs.

Moreover, we have elucidated the physical properties of phenylphosphonium-based *a*_g_(RPh_3_P)[Mn(dca)_3_] glasses and compared that with *a*_g_(TAlA)[Mn(dca)_3_] as well as other inorganic or ZIF-based glasses. They were found to be moderate electrical conductors at room temperature. However, their thermal conductivities are very low and are as such promising for applications such as waste heat energy harvesting thermoelectrics. For example, a series of thermoelectric glasses may be envisaged by tuning the chemistry of A site cation, which could enhance the charge transfer hoping of the anionic charges between the dicyanamide linkers and increase the electrical conductivity. Such a low κ_RT_ will also be very useful for photovoltaic devices in preventing light-deposited heat, which can cause mechanical stress and limit the lifetime of the material. Notably, these amorphous hybrid organic–inorganic networks similar to the amorphous CPs, owing to their intriguing transport properties, open up new material directions toward energy conversion applications, e.g., fuel cell. Overall, this study provides a rationale for altering the physical properties of hybrid organic–inorganic materials, and at the same time opens up directions for forming further examples of functional liquids and glasses from hybrid structures.

## Materials and Methods

### PXRD

#### Ambient Temperature

X-ray powder diffraction (PXRD) patterns were recorded (2θ = 5–60°) on a Bruker D8 Advance diffractometer (equipped with a LynxEye EX linear position sensitive detector) in Bragg–Brentano geometry using a Cu Kα (λ = 1.540598 Å) source fitted with a Ni 0.012 mm filter. Data were collected using a 2θ step size of 0.02° with 10 s per step. Pawley refinement was carried out using TOPAS academic v6 software (Figure S1).^59^

#### Variable Temperature

The experiments were conducted using an Empyrean Panalytical (Cu Kα source, λ = 1.540598 Å) with a PIXcel detector in 1D scanning mode. The Bragg–Brentano geometry was used with a 10 mm length-limiting slit and a 2.5° Soller slit at the incident section, a 2.5° Soller slit with a Kβ filter, and a programmable antiscattering slit in the receiving part. A powder sample (∼200 mg) was placed on a corundum holder and installed in an Anton Paar XRK 900 reaction chamber, which was connected to the XRD instrument. A continuous Nitrogen flow of 16 mL min^–1^ was used during the whole experiment. The heating stage was connected to a chiller, where continuous flow of water was maintained to achieve fast cooling. Diffraction patterns were collected with a step size of 0.02° at a rate of 10° min^–1^.

### Thermal Analysis

To determine the temperature of decomposition, thermogravimetric analysis was carried out in an SDT apparatus (TA Q600). Data were collected in the range from 25 to 450 °C at a scan rate of 10 °C min^–1^ under an argon atmosphere. To obtain the liquid states, samples (∼10 mg) were placed into a 70 μL alumina crucible and heated above their respective melting offsets at the same heating rate. DSC measurements were conducted using a TA Q2000 and a Netzsch 214 Polyma instrument.

### Preparation of Glasses

#### *a*_g_(MePh_3_P)[Mn(dca)_3_]

The (MePh_3_P)[Mn(dca)_3_] single crystal was heated at 10 °C min^–1^ to 270 °C, then cooled under an argon atmosphere (flow rate 50 mL min^–1^) to −50 °C at ca. 10 °C min^–1^ to obtain glass (Figures S3 and S4).

#### *a*_g_(EtPh_3_P)[Mn(dca)_3_]

The (EtPh_3_P)[Mn(dca)_3_] single crystal was heated at 10 °C min^–1^ to 230 °C, then cooled under an argon atmosphere (flow rate 50 mL min^–1^) to −50 °C at ca. 10 °C min^–1^ to obtain glass (Figures S3 and S4).

#### *a*_g_(Ph_4_P)[Mn(dca)_3_]

The (Ph_4_P)[Mn(dca)_3_] single crystal was heated at 10 °C min^–1^ to 270 °C, then cooled under an argon atmosphere (flow rate 50 mL min^–1^) to −50 °C at ca. 3 °C min^–1^ to obtain glass (Figures S3 and S4).

### Nuclear Magnetic Resonance (NMR)

#### Solid-State NMR

^1^H, ^31^P, and ^13^C experiments were performed on a 9.4 T Bruker Avance III HD spectrometer equipped with a 1.3 mm HXY MAS probe in double resonance. Additionally, ^1^H spectra were also recorded on an 18.8 T Bruker Avance Neo spectrometer equipped with a 1.3 mm HX MAS probe, as the increase in external magnetic field strength results in enhanced spectral resolution. The ^1^H channel was tuned to ν_0_(^1^H) = 400.13 MHz at 9.4 T and ν_0_(^1^H) = 800.30 MHz at 18.8 T, while the X channel was tuned to ν_0_(^31^P) = 161.98 MHz or ν_0_(^13^C) = 100.61 MHz at 9.4 T. All spectra were recorded under a MAS frequency of ν_*r*_ = 60 kHz, corresponding to a sample temperature of at least ∼50–55 °C, as measured from the temperature dependence of the ^79^Br NMR chemical shift and spin–lattice relaxation time constant (*T*_1_) of KBr.^60^ Pulses were applied at a radio frequency (rf) field amplitude of ν_1_ = 200 kHz and ^13^C and ^31^P experiments were performed without ^1^H decoupling. MAS NMR spectra were recorded using a double adiabatic echo pulse sequence consisting of a square π/2 excitation pulse of length equal to 1.25 μs followed by two rotor-synchronized short, high-power, adiabatic pulses (SHAPs) of length equal to 50 μs sweeping through 10 MHz and designed to achieve efficient population inversion in paramagnetic solids.^61^^1^H ^13^C double-resonance 1D TEDOR experiments were performed to facilitate the spectral assignment as only signals corresponding to protonated carbons are observed in these spectra.^61,62^ An optimized recoupling time of 50 μs (i.e., 3 rotor periods) was used and adiabatic SHAPs inversion pulses were applied to the ^1^H channel to enhance the polarization transfer.^61^^1^H, ^31^P, and ^13^C double adiabatic echo spectra were recorded with a recycle delay of at least 5 times the *T*_1_ value of the corresponding nucleus and 256, 20 480, and 512 000 scans, respectively. ^13^C TEDOR experiments were recorded with 4 096 000 scans and a recycle delay of at least 1.3 times the ^1^H *T*_1_ to maximize the signal-to-noise ratio per time unit. ^1^H, ^31^P and ^13^C spectra are reported relative to the ^1^H signal of adamantane at 1.85 ppm,^63^ the ^31^P signal of 85 wt % H_3_PO_4_ in H_2_O at 0.00 ppm and the tertiary ^13^C signal of adamantane at 29.45 ppm,^64^ respectively.

#### Liquid-Phase NMR

Experiments were performed on a 9.4 T Bruker Avance III HD spectrometer equipped with a 5 mm BBFO probe. 8 mg of sample was digested in 100 μL of 35 wt % DCl in D_2_O, and the mixture was dissolved in 500 μL of DMSO-*d*_6_. Additionally, 50 μL of 85 wt % H_3_PO_4_ in H_2_O was added to reference the ^31^P spectra. ^1^H chemical shifts are reported relative to the ^1^H signal corresponding to the residual protons in DMSO-*d*_6_ at 2.50 ppm,^65^ while ^31^P and ^13^C chemical shifts are referenced to the ^31^P H_3_PO_4_ signal at 0.00 ppm and the ^13^C DMSO-*d*_6_ signal at 39.50 ppm,^65^ respectively. ^31^P and ^13^C experiments were performed with ^1^H decoupling. ^1^H, ^31^P, and ^13^C spectra were recorded with 196, 600, and 3840 scans, respectively.

### High-Resolution Mass Spectrometry (HRMS)

Experiments were performed on an Agilent 6540A quadrupole-time-of-flight (TOF) mass spectrometer using electrospray ionization (ESI) in either negative or positive mode. Digested samples were prepared by dissolving either ∼1 mg of crystalline sample in CH_3_OH or ∼2 mg of glassy sample in a mixture of 0.5–1 mL of CH_3_OH, 0.3–0.5 mL of (CH_2_)_4_O, and 0.1 mL of HCO_2_H to enhance sample solubility. A 20:80 mixture of 0.1% HCO_2_H in H_2_O and CH_3_OH was used as the eluent.

### X-ray Total Scattering Experiments

Room-temperature measurements were performed on a sample of crystalline and melt-quenched glass of (RPh_3_P)[Mn(dca)_3_]. Data were collected at the I15-1 beamline at the Diamond Light Source, U.K. (λ = 0.158345 Å, 78.3 keV) in the range 0.6 < *Q* < 24 Å^–1^. Finely ground samples of the crystals and glasses were loaded into 1 mm-diameter borosilicate glass capillaries under Argon atmosphere, and capped with glue to keep the powder in place during the data collection. Data on the empty instrument and capillary were also collected in the same region of 0.6 < *Q* < 24 Å^–1^. Background, multiple scattering, container scattering, Compton scattering, and absorption corrections were performed using the GudrunX program.^66^

### Refinement of Structures against Pair Distribution Function Data and Calculation of Partial Pair Distribution Functions

Published structural models were refined against PDF data using PDFGui in the range 0.5 < *r* < 20 Å, with *Q*_max_ = 22 Å^–1^.^67^ Starting values used were: *Q*_damp_ = 0.08, *S*_ratio_ = 1, and model scale factor = 1.0. Values set and not refined were *r*_cut_ = 5.75 Å, data scale factor = 0.5, and *Q*_broad_ = 0.0001. Isotropic thermal parameters were used for all atoms and initially set to the same value of 0.003 Å^2^.

The published structures of (RPh_3_P)[Mn(dca)_3_] included positional disorder in the dca anion and in the A site molecule, modeled by partial occupancies of multiple sites. To enable refinement, only one of each multiple-site option was chosen to give chemically sensible linkers and cations. The chosen sites were assigned full occupancy and all other sites were discarded. The final structural model was consistent with the given chemical formula. The atomic positions of the dca linkers were refined with appropriate symmetry constraints, and no positions appeared significantly different from their starting ones. The atomic positions of the A cations were not refined, given the substantial disorder of this site. Four distinct thermal parameters for Mn, C, H, and N were refined isotropically. Note that a good fit using this model is not expected; the disorder in the published structure strongly implies that the positions of the ions will vary from one unit cell to another. It is not possible for PDFGui to accurately account for these differences using a “small box” model based on a single unit cell and this is reflected in the relatively poor fits.

### Network Density Measurements

The physical densities of all crystals and glasses were measured using a Micromeritics Accupyc 1340 helium pycnometer. The typical mass used for each test was around 80 mg. The reported values were averaged over a cycle of 10 measurements.

### Magnetic Study

A SQUID MPMS 3 instrument was used to conduct magnetic measurements of (RPh_3_P)[Mn(dca)_3_] crystals and glasses. Details regarding the sample preparation are given in the Supporting Information.

### Elemental Analysis

The determination of the CHN-values (simultaneously) is based on combustion/GC analysis with a EuroEA Elemental Analyzer (made by HEKAtech). Error range is ±0.3%. After acidic digestion and preparation for photometric measurement, Phosphorus is determined by absorption at λ = 410 nm (Cary 100 UV/vis-photometer made by Agilent). Error range is ±0.5%.

### FT-IR Study

Fourier transform Infrared spectra were collected in Transmittance mode using a Bruker Tensor 27 spectrometer on crystal prior to melting and glass samples.

### Nanoindentation

Dense quenched pieces of glass were mounted with epoxy resin and finely polished prior to Nanoindentation tests. An MTS Nanoindenter XP instrument was used under dynamic displacement-controlled mode, at a constant strain rate of 0.05 s^–1^ at ambient conditions. Deformation of the polished samples was made using a Berkovich diamond tip, precalibrated with fused silica. A Poisson’s ratio of ν = 0.2 was used in accordance with prior literature. Values of hardness (*H*) were determined from the variable indentation depth scans to a maximum surface penetration of 500 nm.

### AC Electrical Conductivity Measurements

Room-temperature AC impedance measurements were carried out using a Solartron 1260 impedance/gain-phase analyzer operating between 1 and 2 × 10^7^ Hz and with an applied voltage of 50 mV. Details regarding the measurement setup are given in the Supporting Information.

### Thermal Conductivity Measurements

The thermal conductivity of the glasses was measured by Quantum Design’s Physical Property Measurement System (DynaCool) using the thermal transport option (TTO) in a two-probe lead configuration. Details regarding the sample preparation and measurement setup are given in the Supporting Information.

## Supporting Information Available

Synthesis and characterization details, NMR and HRMS data, MAS NMR data of the crystalline phases, magnetic measurement details, electrical conductivity and thermal conductivity measurement details, PXRD, DSC, and TGAs, FT-IR, synchrotron X-ray total scattering data, and calculated total PDF data (PDF)
