# Supplementary material for: (RPh3P)[Mn(dca)3]: A Family of Glass-Forming Hybrid Organic–Inorganic Materials
Source: Inorg Chem. 2024 Dec 19;63(52):24812–24. doi: 10.1021/acs.inorgchem.4c04181 (PMC11688669; doi:10.1021/acs.inorgchem.4c04181)
Supplement: Supplementary file 1 — ic4c04181_si_001.pdf [file ic4c04181_si_001.pdf]

Supporting Information

(RPh$_3$P)[Mn(dca)$_3$]: A Family of Glass-Forming Hybrid Organic–Inorganic Materials

Bikash Kumar Shaw,[*,†,‡] Lucia Corti,[§,∥] Joshua M. Tuffnell,[†,⊥] Celia Castillo-Blas,[†] Patrick Schlachta,[‡] Georgina P. Robertson,[†] Lauren McHugh,[†,§] Adam F. Sapnik,[†] Sebastian A. Hallweger,[‡] Philip A. Chater,[#] Gregor Kieslich,[‡] David A. Keen,[∇] Sian E. Dutton,[⊥] Frédéric Blanc,[§,∥,○] Thomas D. Bennett[†]

[†] Department of Materials Science and Metallurgy, University of Cambridge, Cambridge CB3 0FS, U.K.

[‡] Department of Chemistry, Technical University of Munich, 85748 Garching, Germany

[§] Department of Chemistry, University of Liverpool, Crown Street, Liverpool L69 7ZD, U.K.

[∥] Leverhulme Research Centre for Functional Materials Design, Materials Innovation Factory, University of Liverpool, Liverpool L7 3NY, U.K.

[⊥] Department of Physics, University of Cambridge, Cambridge CB3 0FS, U.K.

[#] Diamond Light Source Ltd, Diamond House, Harwell Campus, Didcot OX11 0DE, Oxfordshire, U.K.

[∇] ISIS Facility, Rutherford Appleton Laboratory, Harwell Campus, Didcot OX11 0QX, U.K.

[○] Stephenson Institute for Renewable Energy, University of Liverpool, Crown Street, Liverpool L69 7ZF, U.K.

Synthesis

Reagents:

$Mn(NO_3)_2·4H_2O$ (98.5%, Sigma-Aldrich), (MePh$_3$P)Br (98%, Aldrich), (EtPh$_3$P)Br (98%, Aldrich), (Ph$_4$P)Br (98%, Aldrich) and Na(dca) (96%, Sigma-Aldrich) were purchased as indicated and used as received.

Procedure:

(MePh$_3$P)[Mn(dca)$_3$] and (EtPh$_3$P)[Mn(dca)$_3$] materials was synthesized by solvent layering. Typically, 10 mL of an aqueous solution (0.502 g, 2 mmol) of the $Mn(NO_3)_2.4H_2O$ was placed at the bottom of a thin crystallization tube, and layered with a mixture of a sodium dicyanamide solution in 10 mL of water (0.534 g, 6 mmol) and the methyltriphenylphosphonium (0.714 g, 2 mmol) and ethyltriphenylphosphonium bromide (0.742 g, 2 mmol) salts in 10 mmol of ethanol respectively. Block-shaped single crystals were isolated from the mother liquor after one week of slow evaporation in an open atmosphere at 298 K. The synthesis of

$(Ph_4P)[Mn(dca)_3]$ is carried out by stirring technique. To do that, Ph_4PBr (0.246 g, 0.5 mmol) in methanol (4 mL) was added to sodium dicyanamide (0.134 g, 1.5 mmol) in an aqueous solution (4 mL). $Mn(NO_3)_2.4H_2O$ (0.128 g, 0.5 mmol) was added, stirred until it was dissolved, and covered. After 15 minutes, tiny rod-shaped chunk of colorless crystals of $(Ph_4P)[Mn(dca)_3]$ started to form, and they were allowed to settle for an hour and then filtered off.[1,2]

Methods

Nuclear magnetic resonance (NMR)

The ^1H MAS NMR spectra of crystalline $(RPh_3P)[Mn(dca)_3]$ (**Figure 6a**) presents either one broad signal or several significantly overlapping signals in the aromatic region which are assigned to the protons of the phenyl rings. In the aliphatic region of the ^1H MAS NMR spectrum of crystalline $(MePh_3P)[Mn(dca)_3]$, one additional signal corresponding to the methyl group appears at ~2.7 ppm. Similarly, two peaks corresponding to the -C***H***$_2$ (~3.4 ppm) and -C***H***$_3$ (~1.4 ppm) units of the ethyl group are present in the ^1H MAS NMR spectrum of crystalline $(EtPh_3P)[Mn(dca)_3]$. No signals are observed in the aliphatic region of the spectrum obtained for crystalline $(Ph_4P)[Mn(dca)_3]$, as expected. The ^{31}P MAS NMR spectra of crystalline $(MePh_3P)[Mn(dca)_3]$ and $(EtPh_3P)[Mn(dca)_3]$ in **Figure 6b** show two ^{31}P signals in a 1:1 ratio, while one peak is observed in the spectrum of crystalline $(Ph_4P)[Mn(dca)_3]$. This is consistent with the number of phosphorous chemical environments in the asymmetric unit cell of the materials. $(MePh_3P)[Mn(dca)_3]$ and $(EtPh_3P)[Mn(dca)_3]$ crystallize with two distinct methyl and ethyl groups in the asymmetric unit cell, respectively. Therefore, as expected, two -***C***H$_3$ signals are resolved in the ^{13}C MAS NMR spectra of crystalline $(MePh_3P)[Mn(dca)_3]$ and $(EtPh_3P)[Mn(dca)_3]$ (**Figure S18**), while the signals corresponding to the two distinct -***C***H$_2$ chemical environments in crystalline $(EtPh_3P)[Mn(dca)_3]$ likely overlap. Several partially overlapping signals are observed in the 150-100 ppm region of the ^{13}C MAS NMR spectra of crystalline $(RPh_3P)[Mn(dca)_3]$ (**Figure 6c**). ^1H ^{13}C transferred-echo double resonance (TEDOR) MAS NMR experiments facilitate the spectral assignments by filtering out the signals corresponding to non-protonated carbons. Signals between 125 and 110 ppm are largely suppressed and are therefore assigned to the quaternary carbons of both the dca^- and RPh_3P^+ units, whilst the remaining signals above 125 ppm are attributed to the -***C***H carbons of the phenyl rings. Notably, the peak line widths observed in the ^1H, ^{31}P and ^{13}C MAS NMR spectra of the crystalline $(RPh_3P)[Mn(dca)_3]$ materials are relatively narrow and some signals appear

in a chemical shift range typical for diamagnetic systems. Both of these behaviors have been previously observed in other paramagnetic Mn(II)-containing materials.[9]

Crystalline $(MePh_3P)[Mn(dca)_3]$

^{1}H NMR: 8.2 ppm (N***H***, dca^-); 7.5 ppm (C***H***, $MePh_3P^+$); 2.8 ppm ($C\boldsymbol{H}_3$, $MePh_3P^+$).

^{31}P NMR: 22.9 ppm ($MePh_3P^+$).

^{13}C NMR: 135.1 ppm ($\boldsymbol{C}_4$, $MePh_3P^+$); 133.1 ppm ($\boldsymbol{C}_2$, $MePh_3P^+$); 130.3 ppm ($\boldsymbol{C}_3$, $MePh_3P^+$); 119.3 ppm ($\boldsymbol{C}_1$, $^1J_{CP}$ ~ 87.1 Hz, $MePh_3P^+$).

$a_g(MePh_3P)[Mn(dca)_3]$

^{1}H NMR: 8.0 ppm (N***H***, dca^-); 7.4 ppm (C***H***, $MePh_3P^+$); 2.8 ppm ($C\boldsymbol{H}_3$, $MePh_3P^+$).

^{31}P NMR: 22.6 ppm ($MePh_3P^+$).

^{13}C NMR: ^{13}C NMR: 135.1 ppm ($\boldsymbol{C}_4$, $MePh_3P^+$); 133.0 ppm ($\boldsymbol{C}_2$, $MePh_3P^+$); 130.3 ppm ($\boldsymbol{C}_3$, $MePh_3P^+$); 119.4 ppm ($\boldsymbol{C}_1$, $^1J_{CP}$ ~ 87.9 Hz, $MePh_3P^+$).

Crystalline $(EtPh_3P)[Mn(dca)_3]$

^{1}H NMR: 7.6 ppm (C***H***, $EtPh_3P^+$); 3.4 ppm ($C\boldsymbol{H}_2CH_3$, $EtPh_3P^+$); 1.0 ppm ($CH_2C\boldsymbol{H}_3$, $EtPh_3P^+$).

^{31}P NMR: 27.3 ppm ($EtPh_3P^+$).

^{13}C NMR: 134.4 ppm ($\boldsymbol{C}_4$, $EtPh_3P^+$); 132.8 ppm ($\boldsymbol{C}_2$, $EtPh_3P^+$); 129.7 ppm ($\boldsymbol{C}_3$, $EtPh_3P^+$); 117.3 ppm ($\boldsymbol{C}_1$, $^1J_{CP}$ ~ 86.7 Hz, $EtPh_3P^+$).

$a_g(EtPh_3P)[Mn(dca)_3]$

^{1}H NMR: 8.0 ppm (N***H***, dca^-); 7.6 ppm (C***H***, $EtPh_3P^+$); 3.3 ppm ($C\boldsymbol{H}_2CH_3$, $EtPh_3P^+$); 1.0 ppm ($CH_2C\boldsymbol{H}_3$, $EtPh_3P^+$).

^{31}P NMR: 27.7 ppm ($EtPh_3P^+$).

^{13}C NMR: 134.9 ppm ($\boldsymbol{C}_4$, $EtPh_3P^+$); 133.2 ppm ($\boldsymbol{C}_2$, $EtPh_3P^+$); 130.2 ppm ($\boldsymbol{C}_3$, $EtPh_3P^+$); 117.7 ppm ($\boldsymbol{C}_1$, $^1J_{CP}$ ~ 84.7 Hz, $EtPh_3P^+$).

Crystalline $(Ph_4P)[Mn(dca)_3]$

^{1}H NMR: 7.9 ppm (N***H***, dca^-); 7.7, 7.6 and 7.5 ppm (C***H***, Ph_4P^+).

^{31}P NMR: 23.7 ppm (J_{PC} ~ 89 Hz, Ph_4P^+).

^{13}C NMR: 135.9 ppm ($\boldsymbol{C}_4$, Ph_4P^+); 134.7 ppm ($\boldsymbol{C}_2$, $^3J_{CP}$ ~ 9.0 Hz, Ph_4P^+); 131.0 ppm ($\boldsymbol{C}_3$, $^2J_{CP}$ ~ 11.6 Hz, Ph_4P^+); 117.7 ppm ($\boldsymbol{C}_1$, $^1J_{CP}$ ~ 89.2 Hz, Ph_4P^+).

$a_g(Ph_4P)[Mn(dca)_3]$

^{1}H NMR: 7.8, 7.6 and 7.5 ppm (C***H***, Ph_4P^+).

^{31}P NMR:24.2 ppm (J_{PC} ~ 89 Hz, Ph_4P^+).

^{13}C NMR: 135.9 ppm ($\boldsymbol{C}_4$, Ph_4P^+); 134.6 ppm ($\boldsymbol{C}_2$, $^3J_{CP}$ ~ 9.3 Hz, Ph_4P^+); 130.9 ppm ($\boldsymbol{C}_3$, $^2J_{CP}$ ~ 11.1 Hz, Ph_4P^+); 117.6 ppm ($\boldsymbol{C}_1$, $^1J_{CP}$ ~ 89.3 Hz, Ph_4P^+).

High Resolution Mass Spectrometry (HRMS)

$(MePh_3P)[Mn(dca)_3]$

HRMS (ESI-, CH_3OH) *m/z*: calculated for [$C_2N_3^-$, dca^-] 66.0098, found 66.0102.

HRMS (ESI+, CH_3OH) *m/z*: calculated for [$C_{19}H_{18}P^+$, $MePh_3P^+$] 277.1141, found 277.1143.

$a_g(MePh_3P)[Mn(dca)_3]$

HRMS (ESI-, $CH_3OH + (CH_2)_4O + HCO_2H$) *m/z*: calculated for [$C_2N_3^-$, dca^-] 66.0098, found 66.0099.

HRMS (ESI+, CH_3OH, $(CH_2)_4O$, HCO_2H) *m/z*: calculated for [$C_{19}H_{18}P^+$, $MePh_3P^+$] 277.1141, found 277.1143.

$(EtPh_3P)[Mn(dca)_3]$

HRMS (ESI-, CH_3OH) *m/z*: calculated for [$C_2N_3^-$, dca^-] 66.0098, found 66.0102.

HRMS (ESI+, CH_3OH) *m/z*: calculated for [$C_{20}H_{20}P^+$, $MePh_3P^+$] 291.1297, found 291.1302.

$a_g(EtPh_3P)[Mn(dca)_3]$

HRMS (ESI-, $CH_3OH + (CH_2)_4O + HCO_2H$) *m/z*: calculated for [$C_2N_3^-$, dca^-] 66.0098, found 66.0102.

HRMS (ESI+, CH_3OH, $(CH_2)_4O$, HCO_2H) *m/z*: calculated for [$C_{20}H_{20}P^+$, $MePh_3P^+$] 291.1297, found 291.1299.

$(Ph_4P)[Mn(dca)_3]$

HRMS (ESI-, CH_3OH) *m/z*: calculated for [$C_2N_3^-$, dca^-] 66.0098, found 66.0101.

HRMS (ESI+, CH_3OH) *m/z*: calculated for [$C_{24}H_{20}P^+$, Ph_4P^+] 339.1297, found 339.1301.

$a_g(Ph_4P)[Mn(dca)_3]$

HRMS (ESI-, $CH_3OH + (CH_2)_4O + HCO_2H$) *m/z*: calculated for [$C_2N_3^-$, dca^-] 66.0098, found 66.0101.

HRMS (ESI+, $CH_3OH + (CH_2)_4O + HCO_2H$) *m/z*: calculated for [$C_{24}H_{20}P^+$, Ph_4P^+] 339.1297, found 339.1301.

Magnetic Measurements

The temperature variation of field-cooled susceptibility (*M-T*) data was collected at 100 Oe magnetic field at a temperature range 10 – 298 K. Samples were placed in a light weight homogeneous quartz tube to minimize the background noise and stray field effects. The magnetic data were corrected for the diamagnetic contribution from the quartz sample holder and the intrinsic diamagnetism of the samples by the standard literature using Pascal's constants.[3]

AC Electrical Conductivity Experiments

The values of DC-resistance were extracted from the ǀZǀ vs ω plot. To perform the measurements, glass samples (~ 20 mg) were ground and pressed in the form of a pellet in a 5 mm pellet dye using hydraulic press with an applied force of 2 Tons. The sample pellet was placed in a sample holder in which the sample is sandwiched between two stainless steel blocking electrodes and contact is maintained by spring pressure. The sample dimensions are as follows: electrode surface area = 0.19 cm^2, thickness = 0.09 cm for $a_g(MePh_3P)[Mn(dca)_3]$; electrode surface area = 0.19 cm^2, thickness = 0.06 cm for $a_g(EtPh_3P)[Mn(dca)_3]$; electrode surface area = 0.19 cm^2, thickness = 0.08 cm for $a_g(Ph_4P)[Mn(dca)_3]$. The data were collected in a moisture-free atmosphere.

Thermal Conductivity Measurements

The data were recorded with temperature ranging from 10 to 298 K in a static mode. To perform the measurements, glass samples (~ 20 mg) were ground and pressed in the form of a pellet in a 5 mm pellet dye using hydraulic press with an applied force of 2 Tons. Sample dimensions are: diameter 0.5 cm; thickness 0.06 cm for $a_g(MePh_3P)[Mn(dca)_3]$, 0.03 cm for $a_g(EtPh_3P)[Mn(dca)_3]$, 0.04 cm for $a_g(Ph_4P)[Mn(dca)_3]$). The pellet was sandwiched between two thermally and electrically conducting disk-shaped copper leads. Good contact ensured with highly conducting silver epoxy adhesive (well cured at 50 ºC for 2 hours). The copper leads were then mounted to the puck (sample holder) very carefully. The system was designed with a copper isothermal radiation shield, which screwed into the base of the puck to minimize radiation between the sample and the environment. A copper shield plate was also placed between the sample stage and the PC board sockets to minimize radiation effects. The emissivity of the glasses was considered as unity (the emissivity is not critical because the radiation heat transfer from the sample is negligible). After achieving the isothermal conditions in the sample, a constant current pulse was passed through the heating element and the temperature of the element was recorded simultaneously by recording its voltage change. The TTO system measures thermal conductivity κ by applying heat from the heater shoe in order to create a specified temperature differential (ΔT) between the two thermometer shoes. It dynamically models the thermal response of the sample to the low-frequency, square-wave heat pulse, and expediting data acquisition.

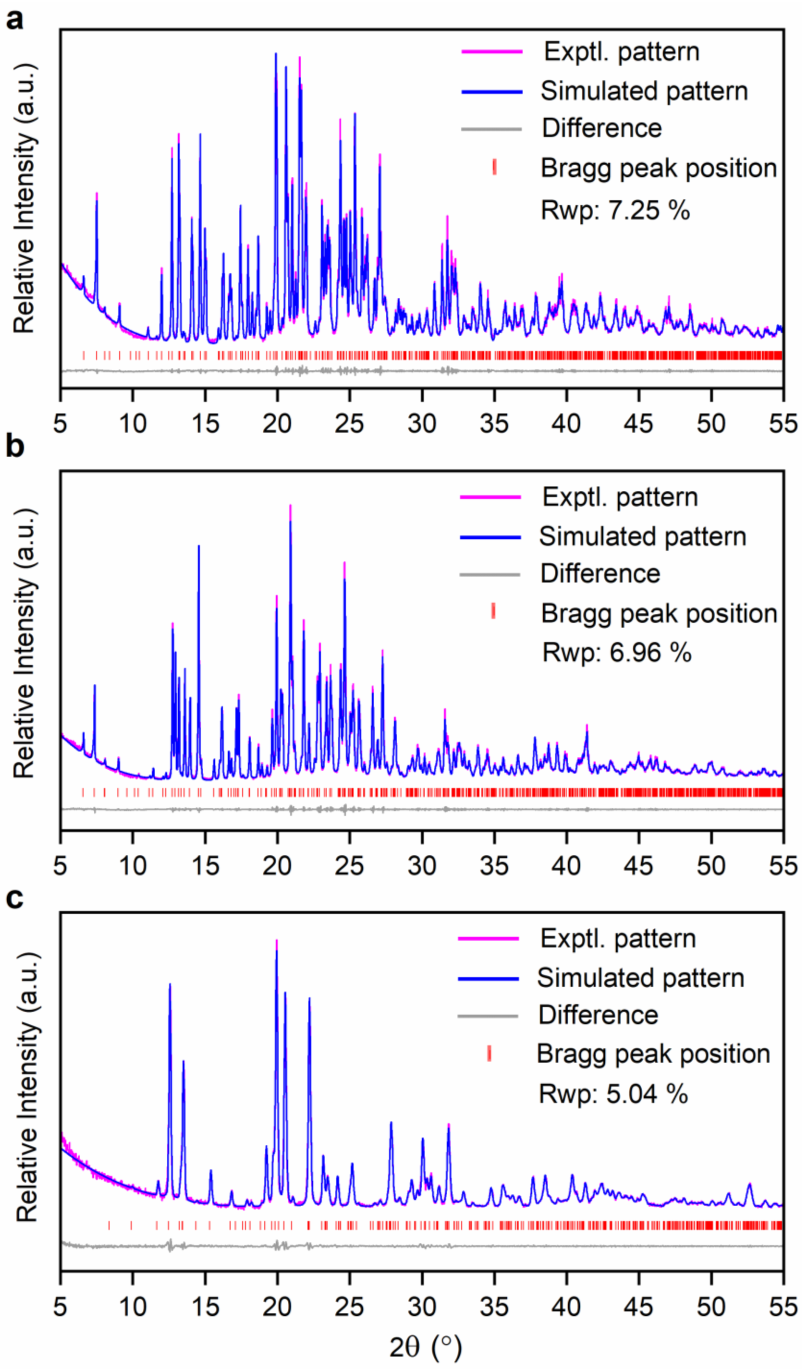

Figure S1: Pawley refinement of X-ray powder diffraction data using TOPAS academic v6 software.[4] **(a)** ($MePh_3P$)[Mn(dca)$_3$], Reported cell parameters (Å): a = 13.9593(3), b = 17.2000(2), c = 20.8587(5), $\alpha = \beta = \gamma = 90°$;[1] Refined cell parameters (Å): a = 14.1435(3), b = 17.2390(4), c = 21.0358(3). **(b)** ($EtPh_3P$)[Mn(dca)$_3$], Reported cell parameters (Å): a = 14.3759(1), b = 16.9055(1), c = 21.6345(2), $\alpha = \beta = \gamma = 90°$;[5] Refined cell parameters (Å): a = 14.4086(2), b = 17.0078(5), c = 21.9217(4). **(c)** (Ph_4P)[Mn(dca)$_3$], Reported cell parameters (Å): a = 13.2564(3), b = 7.5742(1), c = 14.2386(3), $\alpha = \gamma = 90°$, $\beta = 98.73°$;[1] Refined cell parameters (Å): a = 13.4018(4), b = 7.5915(5), c = 14.4005(5), $\alpha = \gamma = 90°$, $\beta = 99.66°$.

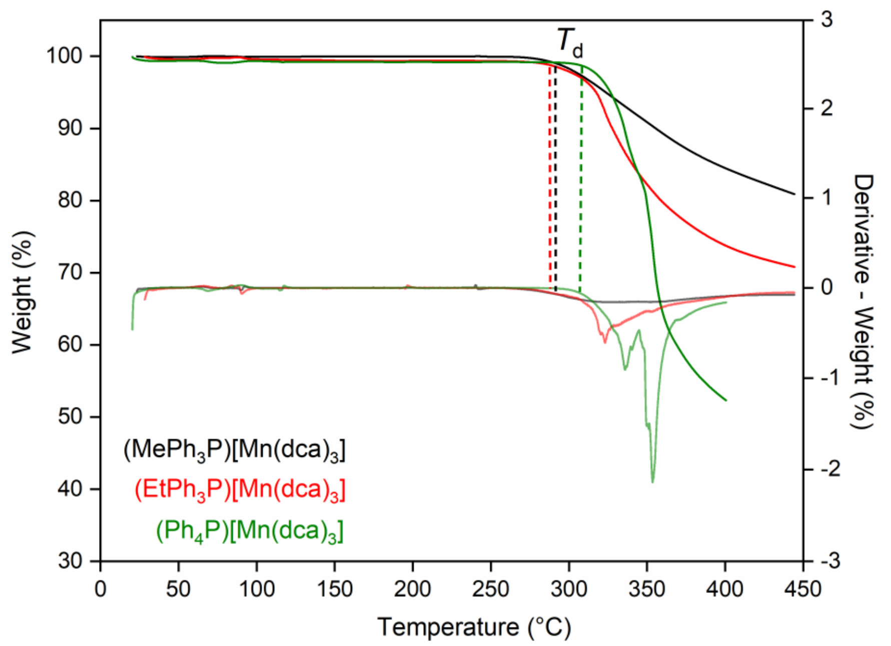

Figure S2: Change in weight % (left axis) and its corresponding first derivative (right axis) were shown with temperature for $(RPh_3P)[Mn(dca)_3]$ (where R = Me, Et, Ph) measured at a rate of 10 °C min^{-1} under an argon atmosphere. The temperature of decomposition (T_d) for each case are indicated by the dashed lines (in respective colors) which follows at 288 °C for $(MePh_3P)[Mn(dca)_3]$, 285 °C for $(EtPh_3P)[Mn(dca)_3]$, 308 °C for $(Ph_4P)[Mn(dca)_3]$ respectively.

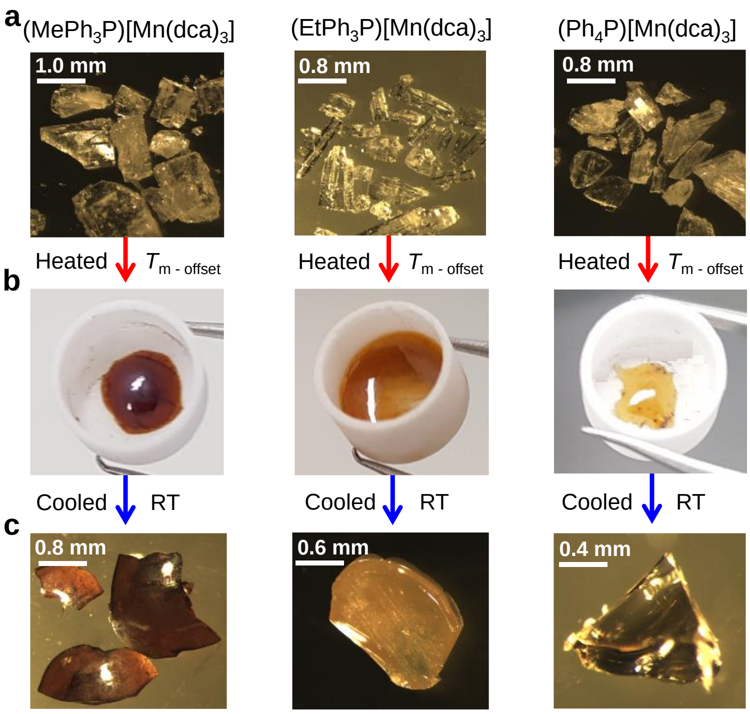

Figure S3: Optical Images of Glass Formation in (RPh_3P)[Mn(dca)$_3$] (where R = Me, Et, Ph) using DSC technique. **(a)** Single crystal samples prior to heating. **(b)** Corresponding liquid appearances immediately after melting upon opening the furnace at $T_{m\text{-offset}}$. Melting was performed in 70-μL alumina crucibles in an Argon atmosphere. (**c**) Glasses formed upon quenching (using the methods described in the manuscript).

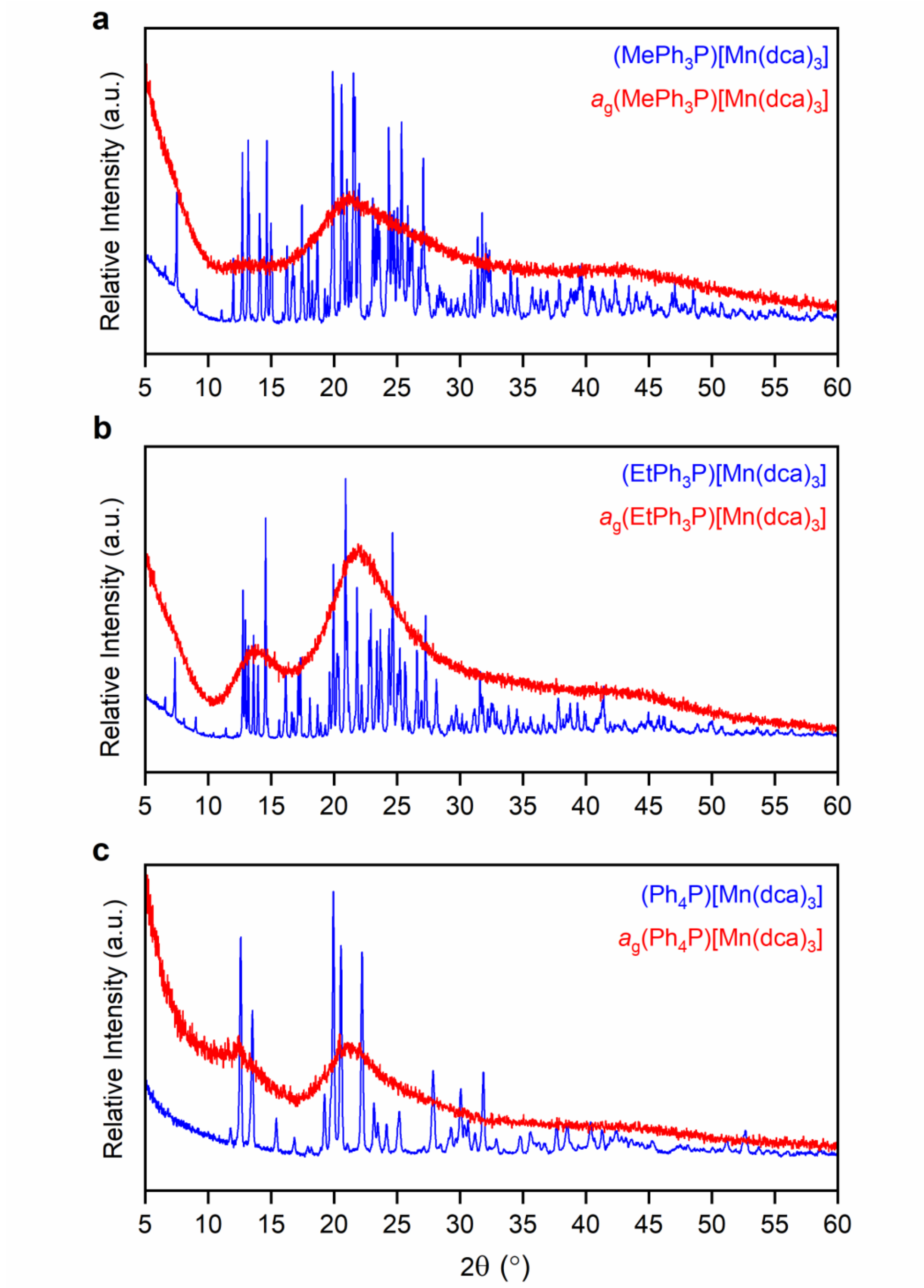

Figure S4: Ambient temperature powder X-ray diffraction patterns of (**a**) $(MePh_3P)[Mn(dca)_3]$, (**b**) $(EtPh_3P)[Mn(dca)_3]$, and (**c**) $(Ph_4P)[Mn(dca)_3]$, before heating (crystal, blue) and upon quenching from the liquid phase (glass, red). These samples correspond to the glasses shown in Supplementary **Figure S3** prepared using the DSC technique.

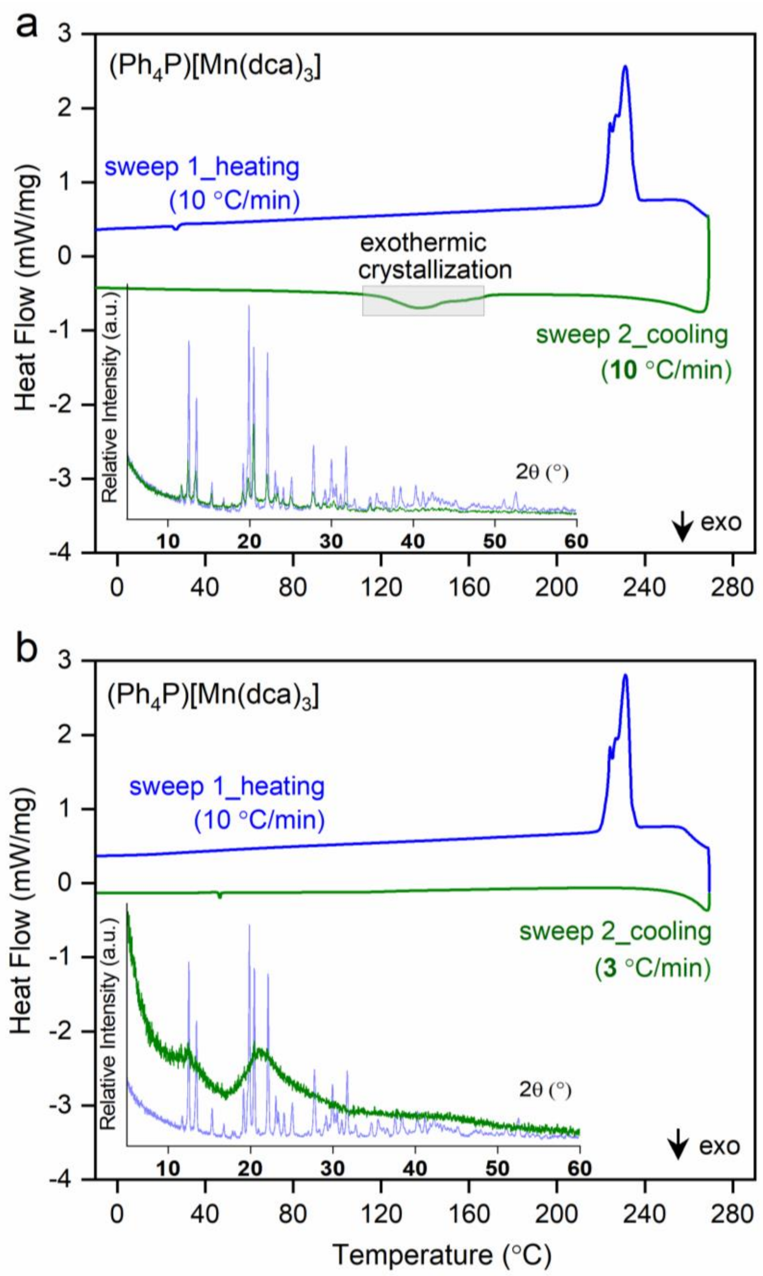

Figure S5: Change in heat flow as a function of temperature for $(Ph_4P)[Mn(dca)_3]$ crystal at a (**a**) heating-cooling rate of 10 °C min^{-1}, (**b**) heating at 10 °C min^{-1} and cooling at 3 °C min^{-1}. While cooling at the same heating rate of 10 °C min^{-1}, the molten liquid show exothermic recrystallization (represented with section under grey box). Inset shows PXRD patterns of the (**a**) recrystallized product and (**b**) amorphous product, after cooling (green). The initial data points upon sweep heating or cooling are omitted for clarity.

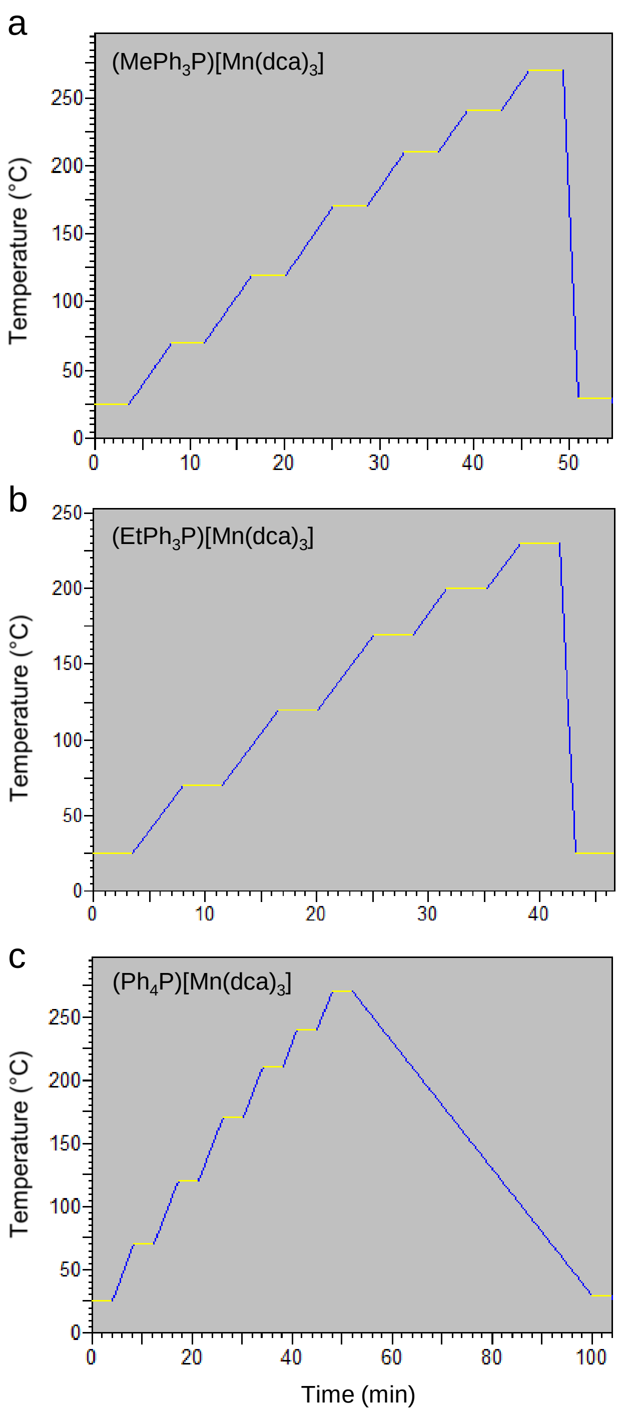

Figure S6. Temperature-Time profiles of variable temperature PXRD experiment for crystalline **(a)** $(MePh_3P)[Mn(dca)_3]$, **(b)** $(EtPh_3P)[Mn(dca)_3]$, and **(c)** $(Ph_4P)[Mn(dca)_3]$. Yellow lines represent 2θ scans from 5° – 30° for $(MePh_3P)[Mn(dca)_3]$, $(EtPh_3P)[Mn(dca)_3]$ and 5° – 33° for $(Ph_4P)[Mn(dca)_3]$ respectively (step size of 0.02° at a rate of 10° min^{-1}). During the heating scans (blue lines), a heating rate of 10 (±1) °C min^{-1} was applied. To comply with the DSC measurements, the maximum instrumental cooling (blue lines) was applied for (a) and (b) which provides a rate of 9 (±1) °C min^{-1}. For (c), we have opted a cooling rate of 5 °C min^{-1} which provides 3 (±1) °C min^{-1}.

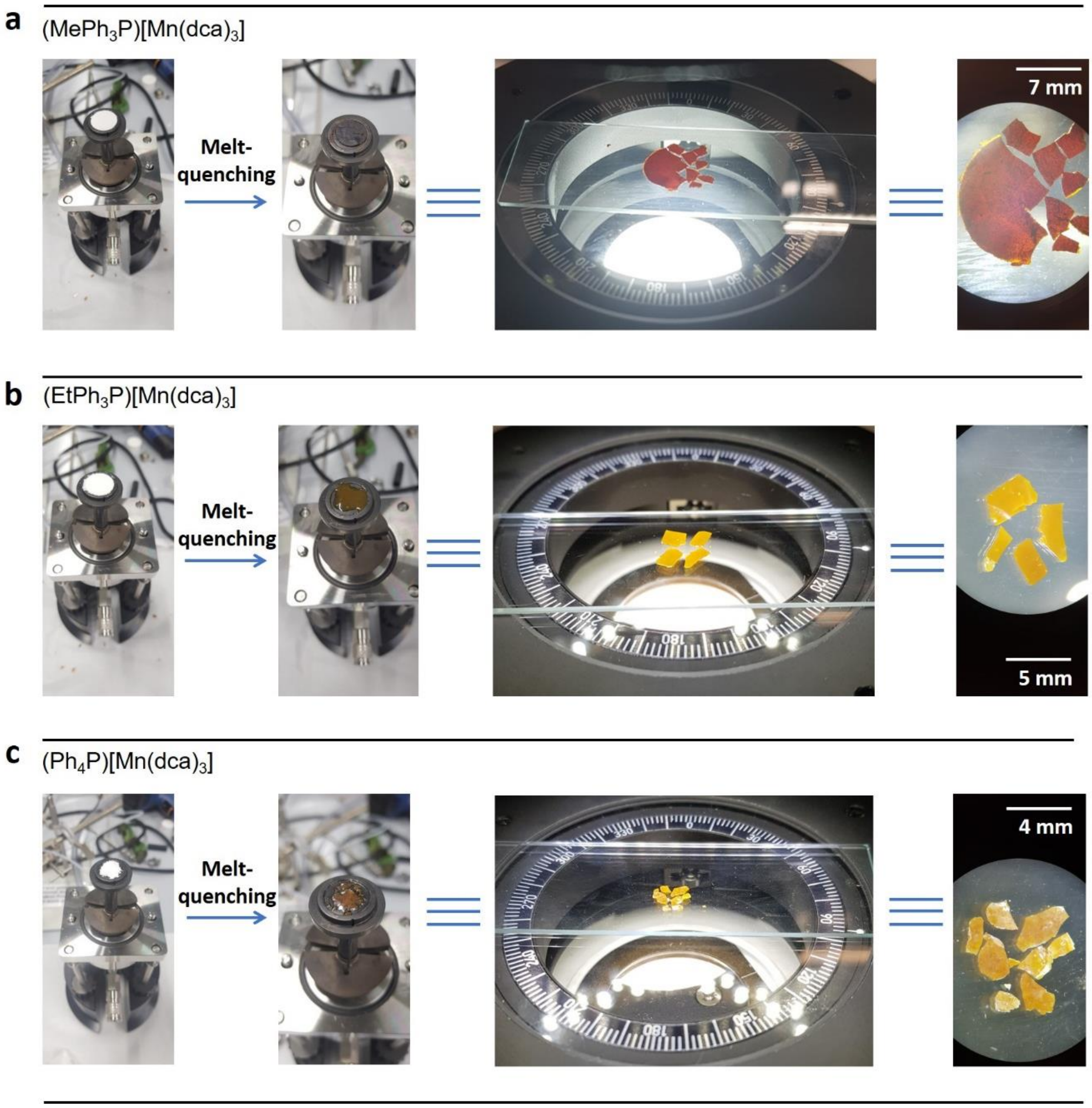

Figure S7. Optical images taken during variable temperature PXRD experiment for **(a)** $(MePh_3P)[Mn(dca)_3]$, **(b)** $(EtPh_3P)[Mn(dca)_3]$, and **(c)** $(Ph_4P)[Mn(dca)_3]$. Ground crystalline powder (~ 200 mg) was placed on the Anton Paar sample holder (diameter 14 mm, thickness 0.8 mm). The second image was obtained after melt-quenching the sample which reveal flow like appearance due to liquification at high temperature. The glass pieces isolated from the sample holder were put under the optical microscope at third and fourth images.

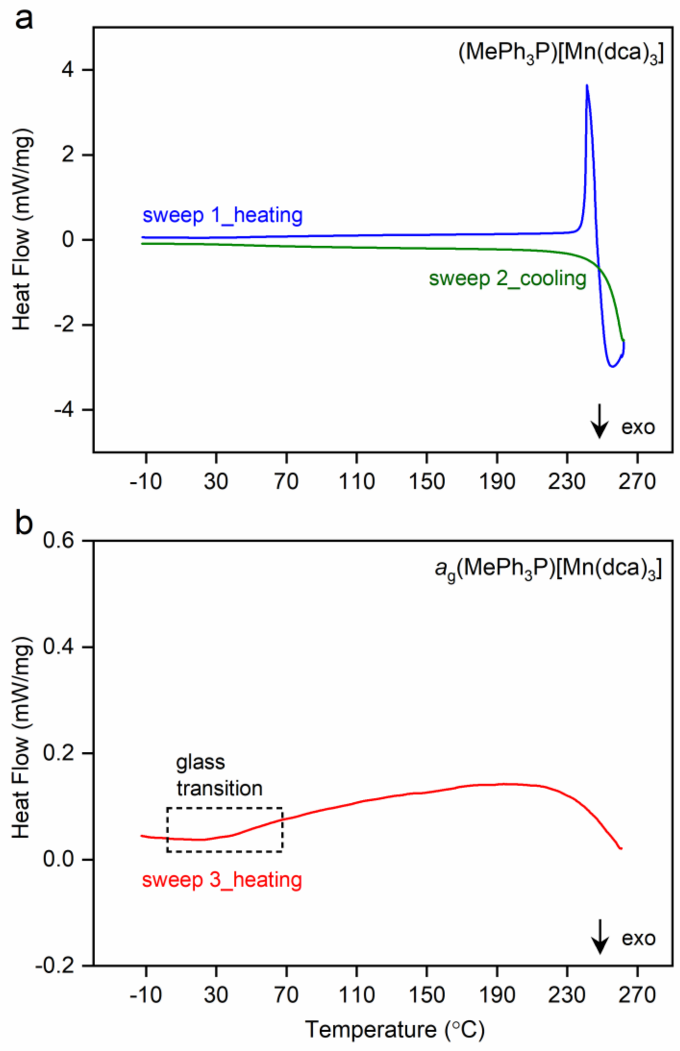

Figure S8: Change in heat flow as a function of temperature for **(a)** $(MePh_3P)[Mn(dca)_3]$ and **(b)** $a_g(MePh_3P)[Mn(dca)_3]$. **(a)** Crystals were first heated above T_m under an argon atmosphere and then cooled to -50 °C at a rate of 10 °C min^{-1}. **(b)** The glass formed after quenching was then reheated at the same heating rate of 10 °C min^{-1} to obtain the glass transition (T_g) which was identified by the upraise in the heat flow and marked with black dashed box. The sharp decrease in the heat flow above 230 °C indicate a partial degradation of the glass. The initial data points upon sweep heating or cooling are omitted for clarity. The experiment was carried out in DSC Q2000 instrument.

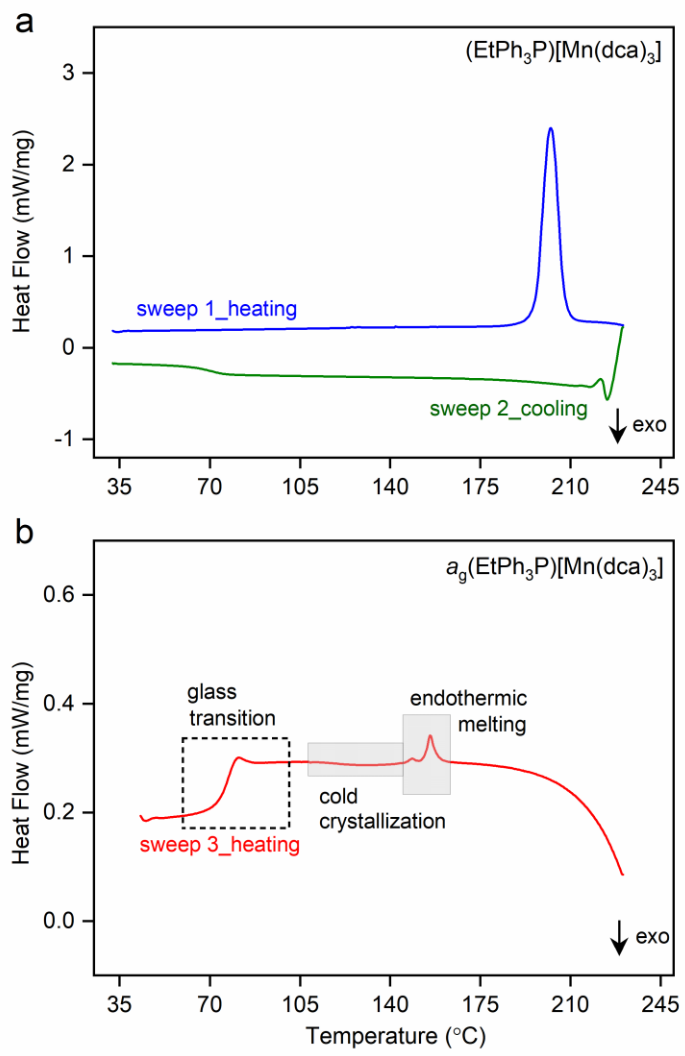

Figure S9: Change in heat flow as a function of temperature for **(a)** $(EtPh_3P)[Mn(dca)_3]$ and **(b)** $a_g(EtPh_3P)[Mn(dca)_3]$. **(a)** Crystals were first heated above T_m under an argon atmosphere and then cooled to 25 °C at a rate of 10 °C min^{-1}. **(b)** The glass formed after quenching was then reheated at the same heating rate of 10 °C min^{-1} to obtain the glass transition (T_g) which was identified by the upraise in the heat flow and marked with black dashed box. While heating, the glass also cold crystallized and then melts at higher temperature (represented with sections under grey boxes). The sharp decrease in the heat flow above 175 °C indicate a partial degradation of the glass. The initial data points upon sweep heating are omitted for clarity. The experiment was carried out in DSC Netzsch 214 Polyma instrument.

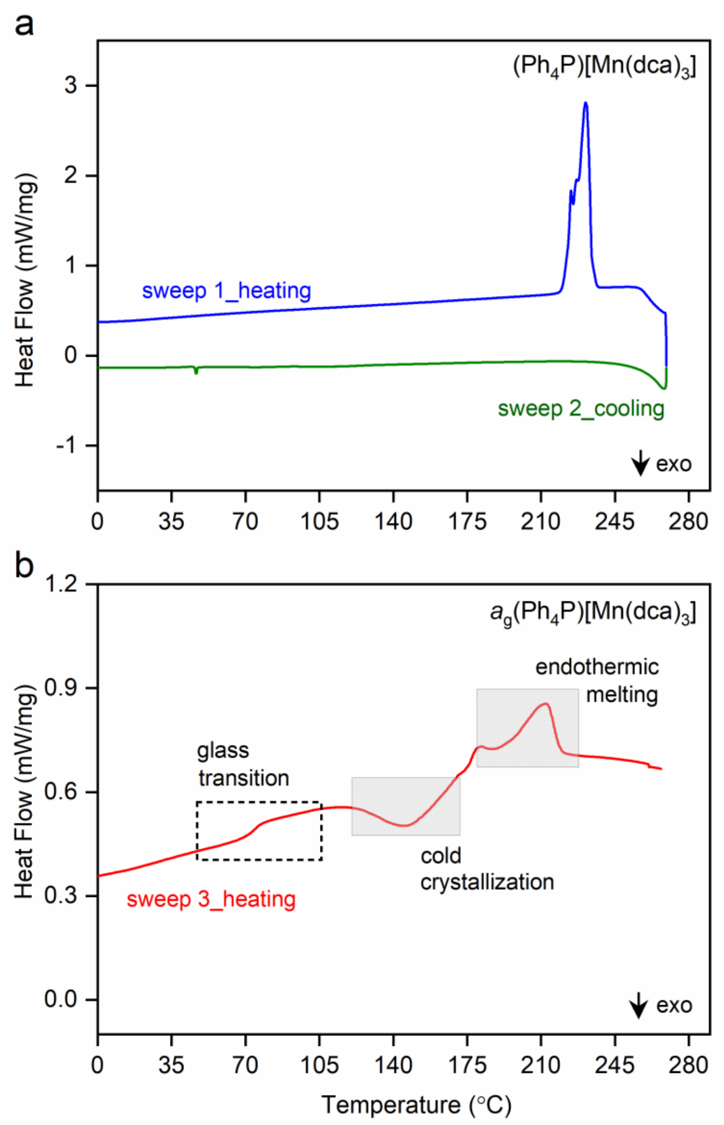

Figure S10: Change in heat flow as a function of temperature for **(a)** $(Ph_4P)[Mn(dca)_3]$ and **(b)** $a_g(Ph_4P)[Mn(dca)_3]$. **(a)** Crystals were first heated above T_m at a rate of 10 °C min^{-1} under an argon atmosphere and then cooled to -30 °C at a rate of 3 °C min^{-1}. **(b)** The glass formed after quenching was then reheated at the same heating rate of 10 °C min^{-1} to obtain the glass transition (T_g) which was identified by the upraise in the heat flow and marked with black dashed box. While heating, the glass also cold crystallized and then melts at higher temperature (represented with sections under grey boxes). The sharp decrease in the heat flow above 230 °C indicate a partial degradation of the glass. The initial data points upon sweep heating or cooling are omitted for clarity. The experiment was carried out in DSC Q2000 instrument.

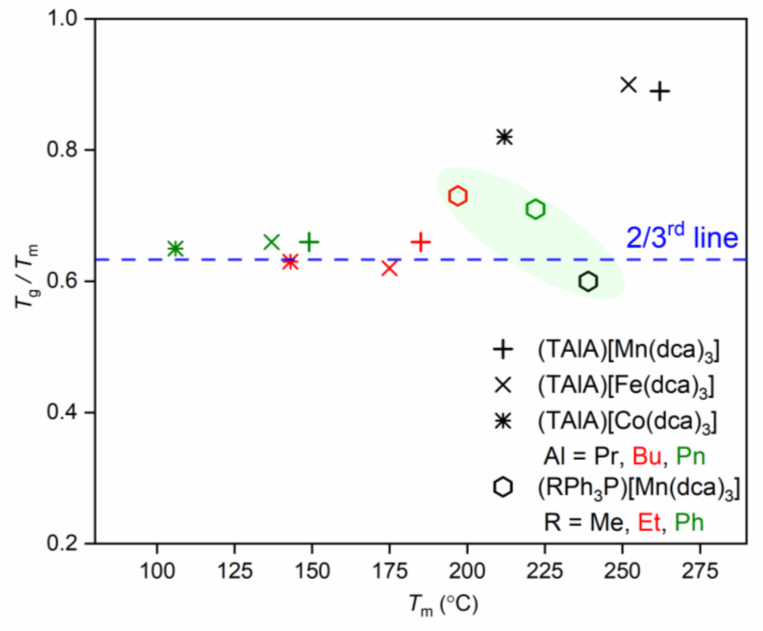

Figure S11: The comparison in T_g/T_m ratio (evaluated in Kelvin units) between several low-melting hybrid organic-inorganic ABX_3 structures.[2] Shaded oval (green) represent the current $a_g(RPh_3P)[Mn(dca)_3]$ series.

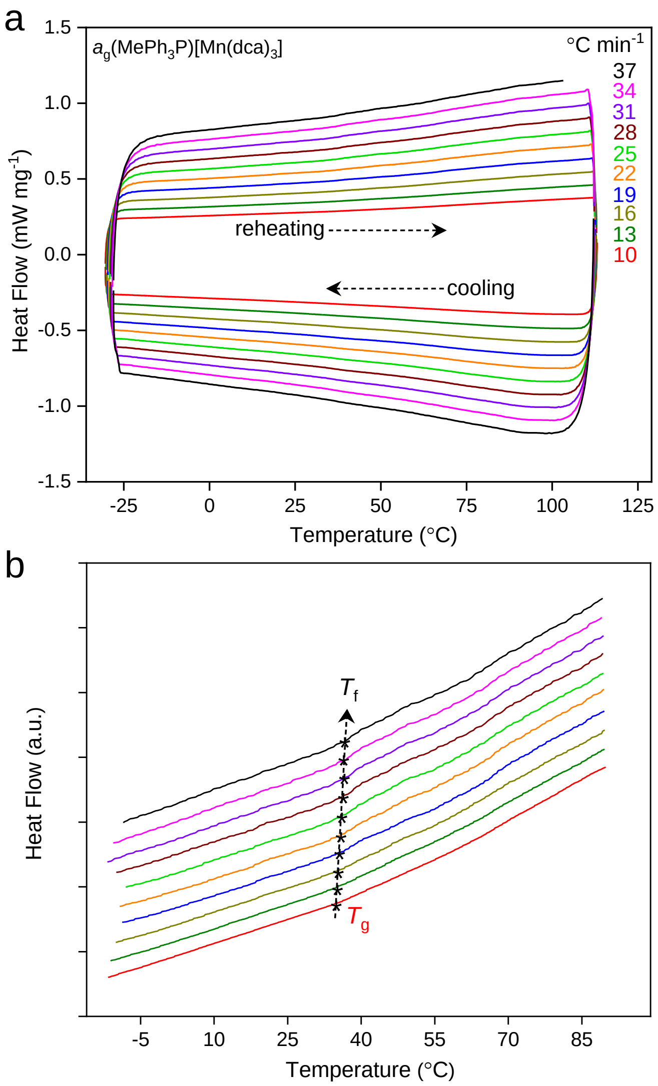

Figure S12. **(a)** Dynamics of DSC cooling-reheating on (MePh$_3$P)[Mn(dca)$_3$] liquid at different rates, allowing determination of the fragility, *m* (as shown in **Figure 5**). **(b)** Zoom-in image of (a) from -16 – 99 °C, from which the T_f (fictive temperature) and T_g (T_f at a rate of 10 K min^{-1}) was graphically derived and marked with asterisk.

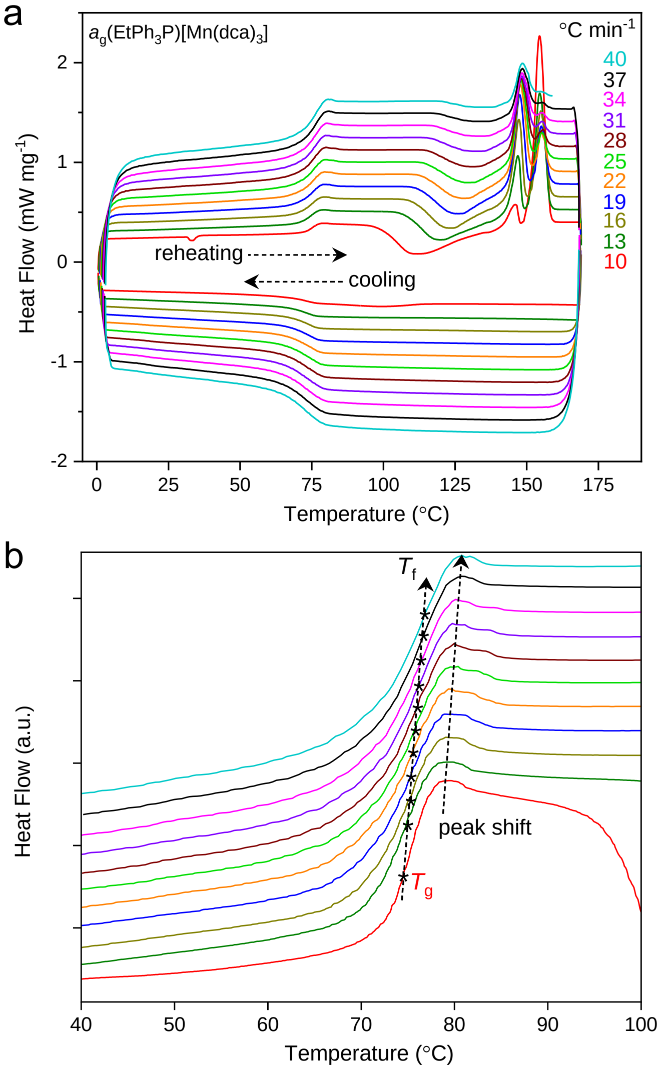

Figure S13. **(a)** Dynamics of DSC cooling-reheating on (EtPh$_3$P)[Mn(dca)$_3$] liquid at different rates, allowing determination of the fragility, *m* (as shown in **Figure 5**). **(b)** Zoom-in image of (a) from 40 – 100 °C, from which the T_f (fictive temperature) and T_g (T_f at a rate of 10 K min^{-1}) was graphically derived and marked with asterisk.

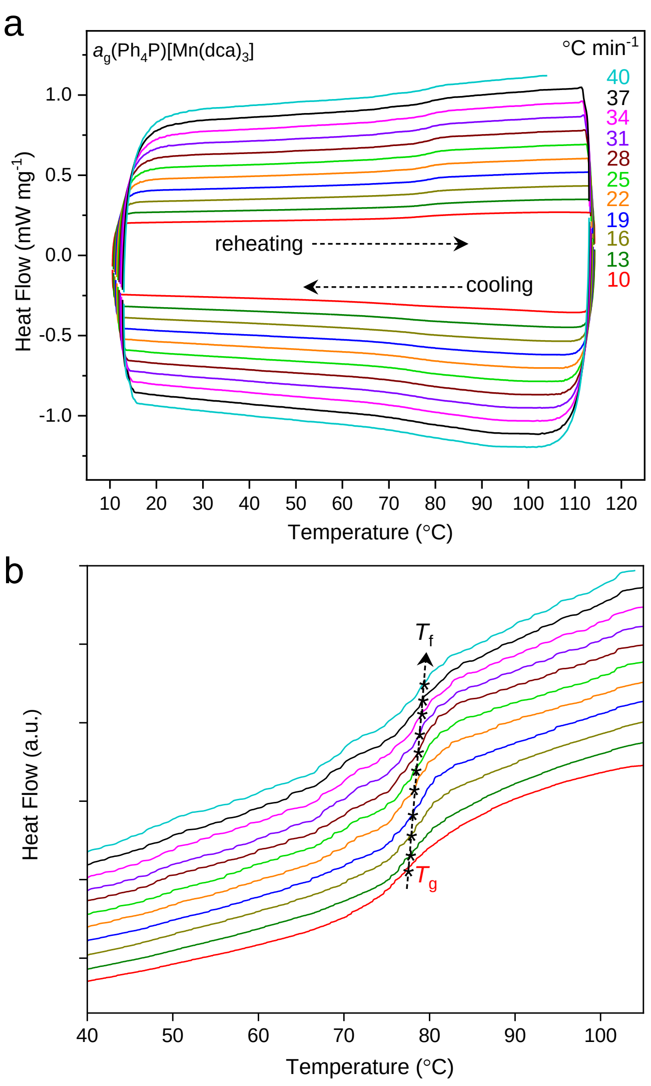

Figure S14. **(a)** Dynamics of DSC cooling-reheating on (Ph_4P)[Mn(dca)$_3$] liquid at different rates, allowing determination of the fragility, *m* (as shown in **Figure 5**). **(b)** Zoom-in image of (a) from 40 – 105 °C, from which the T_f (fictive temperature) and T_g (T_f at a rate of 10 K min^{-1}) was graphically derived and marked with asterisk.

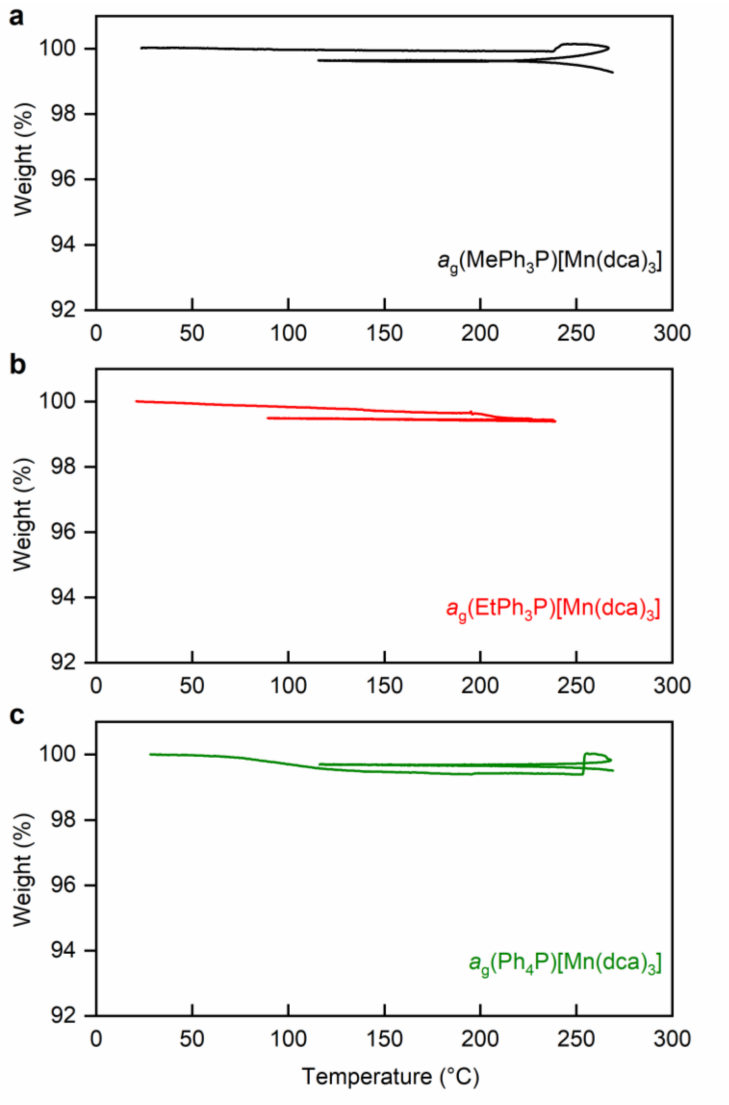

Figure S15: Values of gravimetric weight change (%) with temperature for two consecutive heating upscans for **(a)** a_g(MePh$_3$P)[Mn(dca)$_3$], **(b)** a_g(EtPh$_3$P)[Mn(dca)$_3$], and **(c)** a_g(Ph$_4$P)[Mn(dca)$_3$] under an argon atmosphere. Temperature profile of the scans were above the melting offset (T_m) identified in each case, before returning to low temperature and then heating again above the melting temperature identified previously.

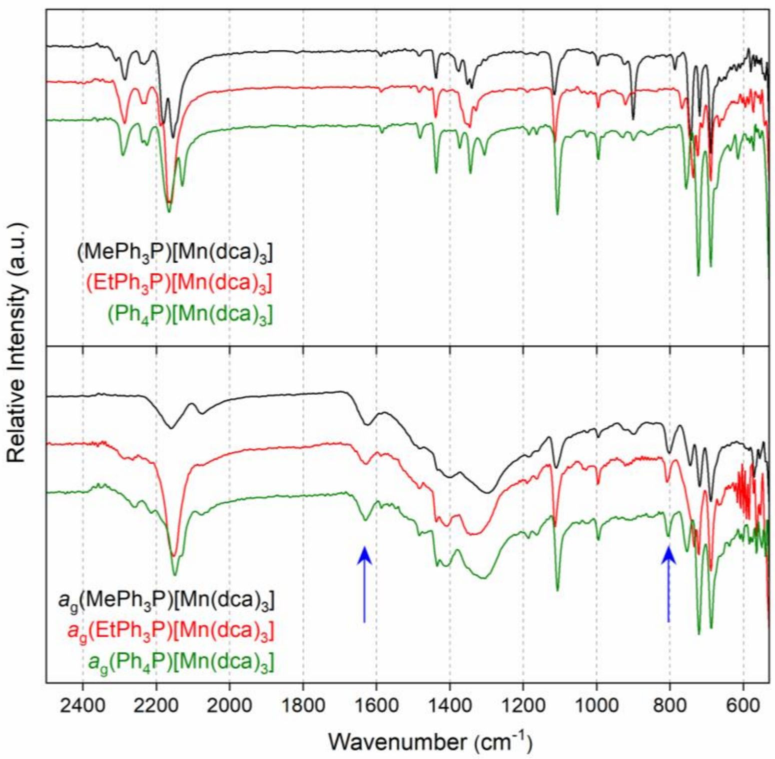

Figure S16: FT-IR spectra of $(RPh_3P)[Mn(dca)_3]$ (where R = Me, Et, Ph), crystals (top) and glasses (bottom). Small bands appeared at 1629-1634 cm^{-1} and 802-806 cm^{-1} regions (indicated by blue arrow) in all $a_g(RPh_3P)[Mn(dca)_3]$ samples indicate high temperature deformation of a portion of dca ligand (vibration of $\delta_{C\text{-}N\text{-}C}$).[6–9]

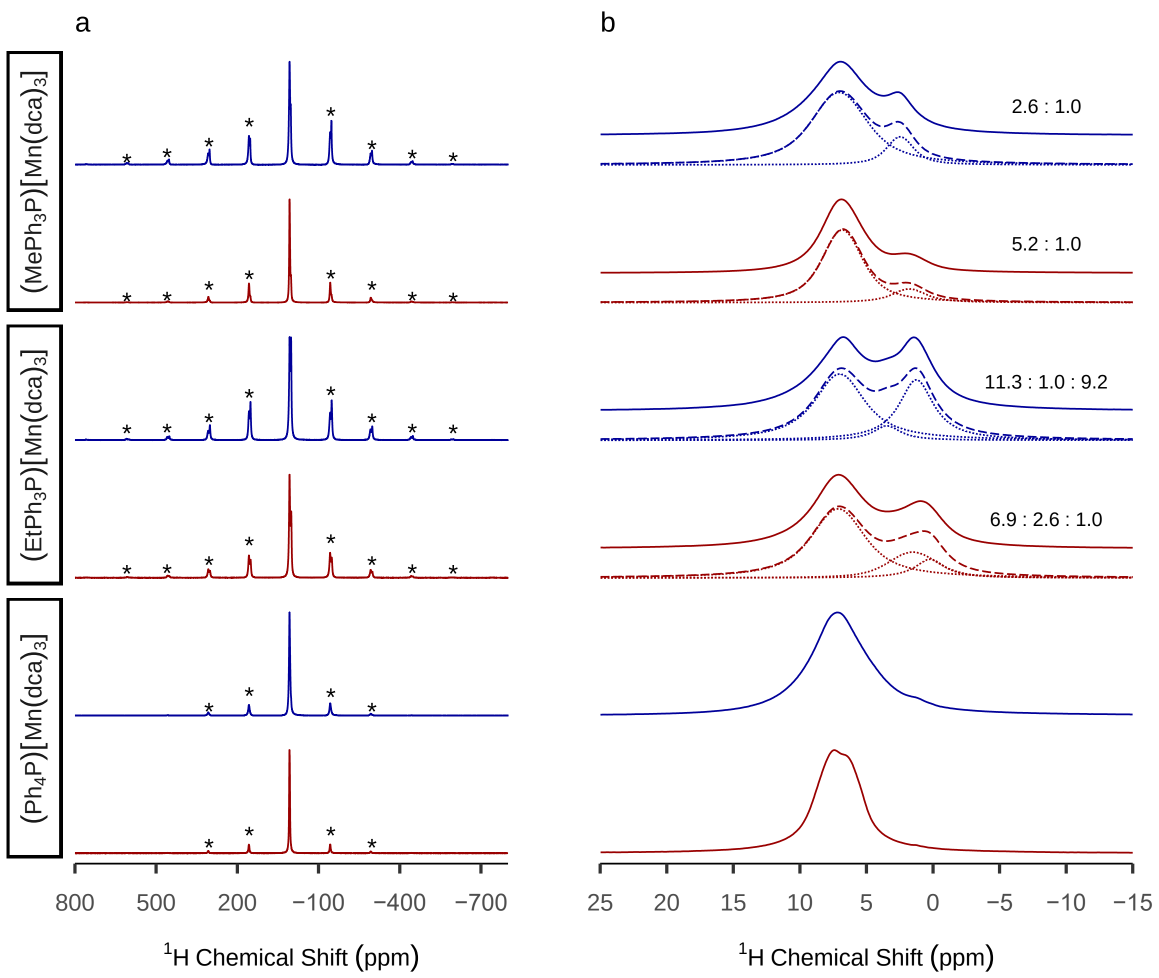

Figure S17: (a) ^{1}H double adiabatic echo MAS NMR spectra recorded at 9.4 T of (blue) crystalline $(RPh_3P)[Mn(dca)_3]$ (where R = Me, Et, Ph) and (red) $a_g(RPh_3P)[Mn(dca)_3]$. Asterisks (*) denote the spinning sidebands. (b) Magnified views of the ^{1}H signals in the spectral region between 25 and -15 ppm. The simulated spectra (dashed lines) and the individual deconvoluted signals (dotted lines) obtained from the line shape analysis are given below the corresponding experimental spectra (continuous lines). The ratios denote the relative area of the deconvoluted signals.

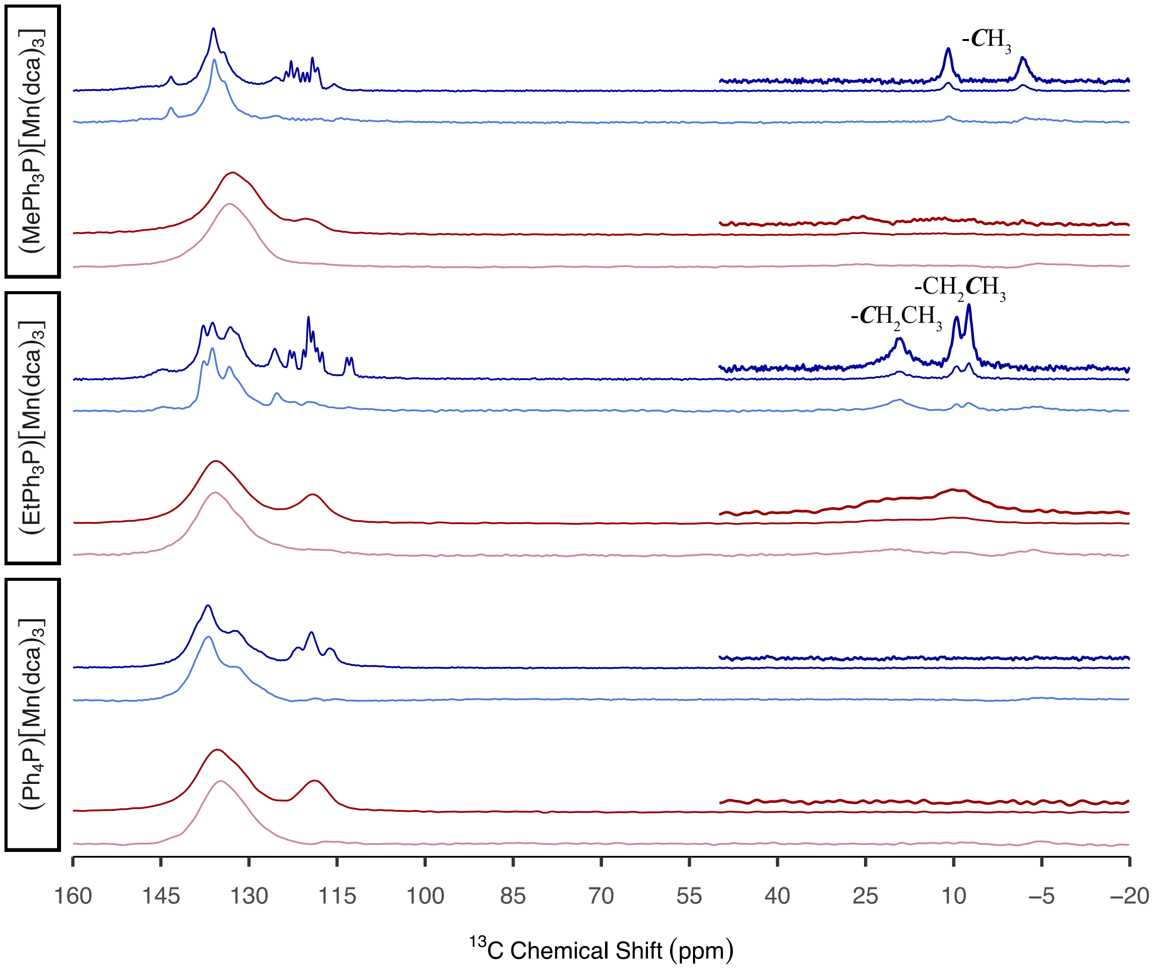

Figure S18: ^{13}C double adiabatic echo MAS NMR spectra of (blue) crystalline $(RPh_3P)[Mn(dca)_3]$ (where R = Me, Et, Ph) and (red) $a_g(RPh_3P)[Mn(dca)_3]$. Magnified views of the 50 to -20 ppm region are shown above the corresponding spectra. ^{1}H ^{13}C TEDOR MAS NMR spectra are shown in lighter shade of blue/red below the corresponding ^{13}C double adiabatic echo MAS NMR spectra. The assignment of the ^{13}C signals observed in the aliphatic region is presented above the crystalline $(RPh_3P)[Mn(dca)_3]$ spectra.

a
b
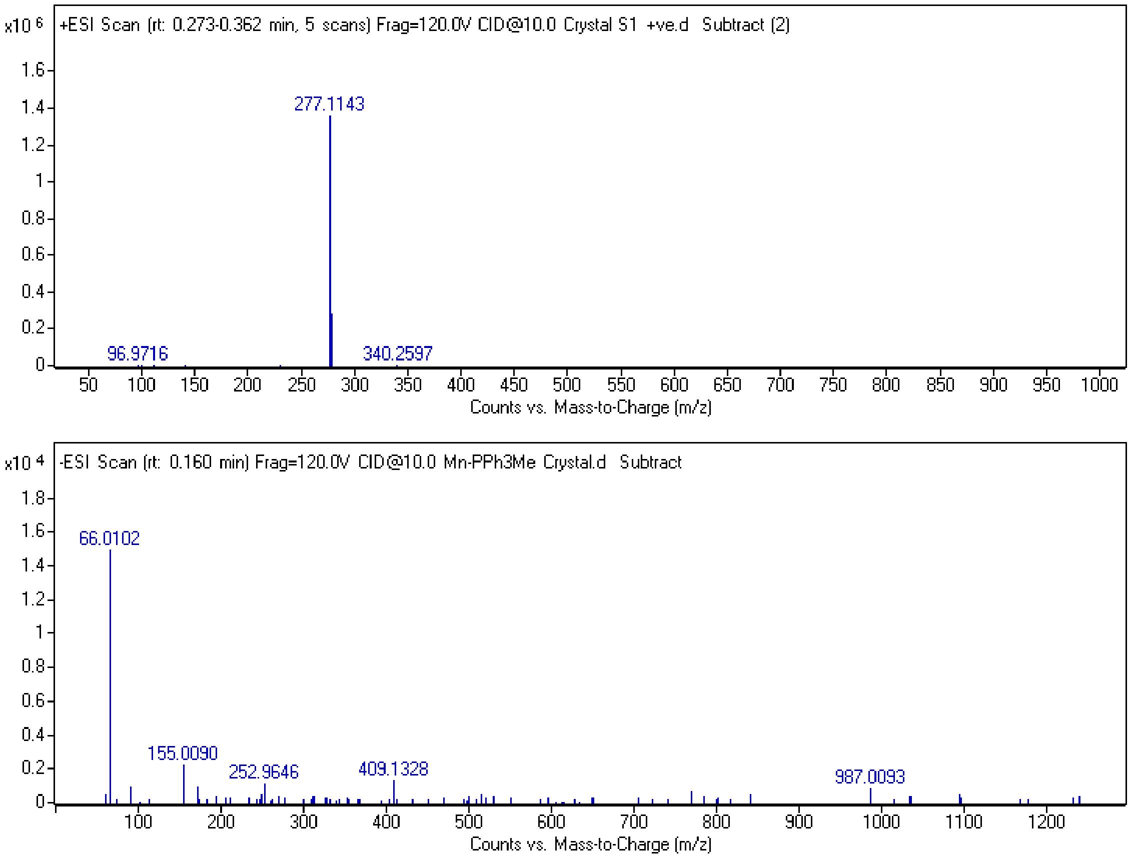

Figure S19: HRMS data of crystalline $(MePh_3P)[Mn(dca)_3]$ with ionisation in (a) positive and (b) negative mode.

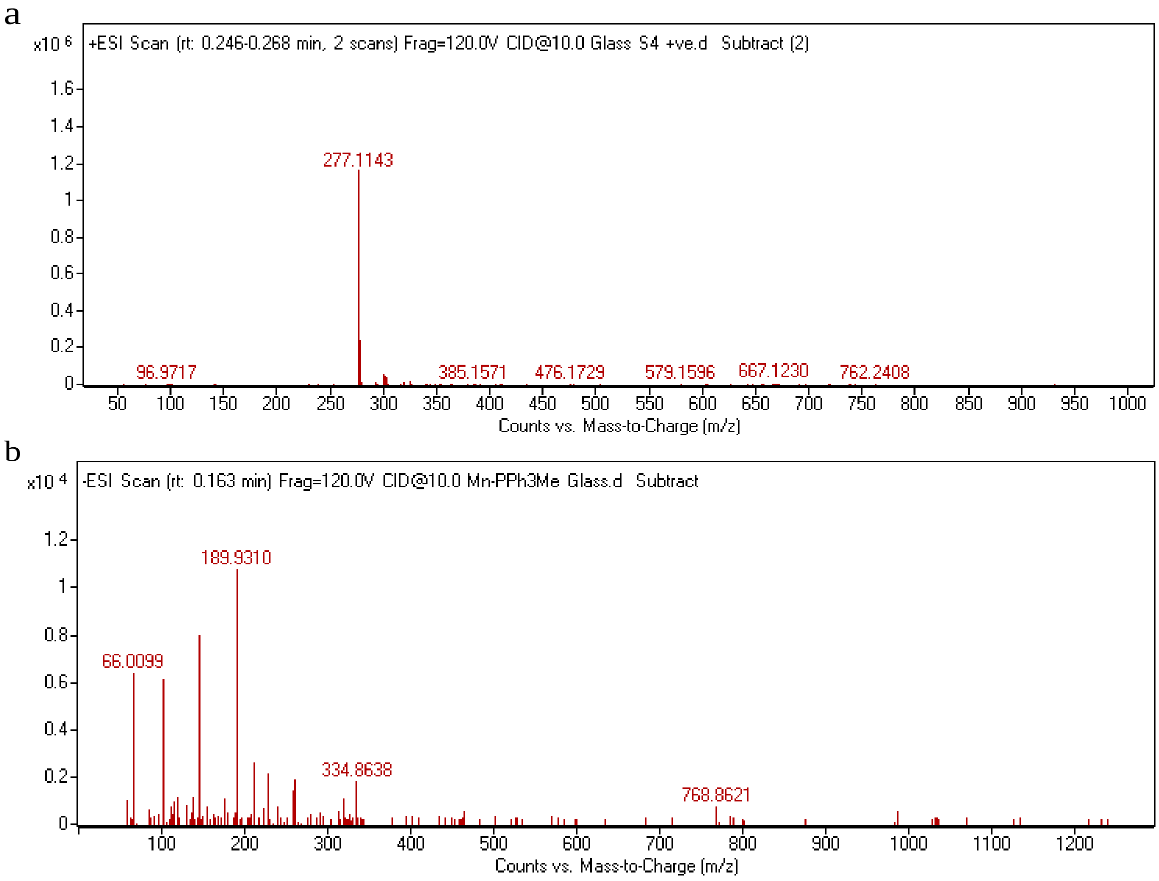

Figure S20: HRMS data of a_g(MePh$_3$P)[Mn(dca)$_3$] with ionisation in (a) positive and (b) negative mode.

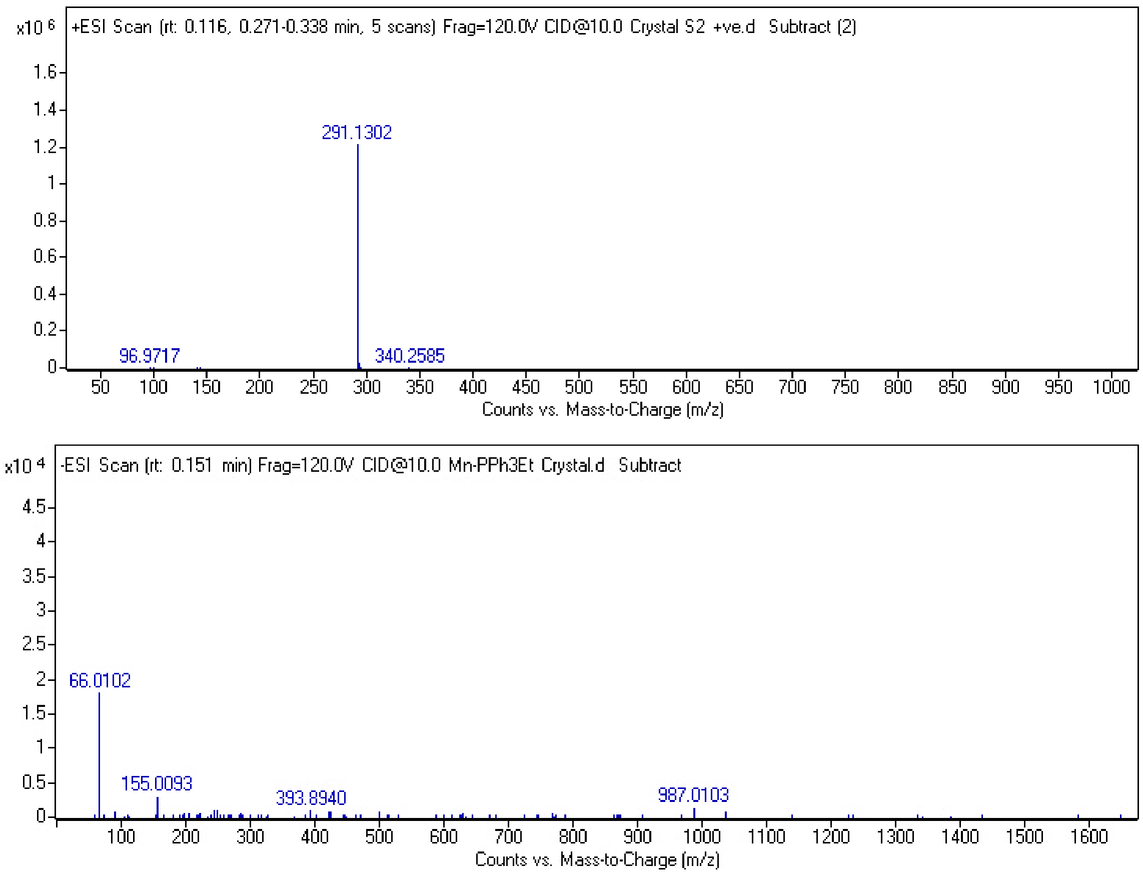

Figure S21: HRMS data of crystalline $(EtPh_3P)[Mn(dca)_3]$ with ionisation in (a) positive and (b) negative mode.

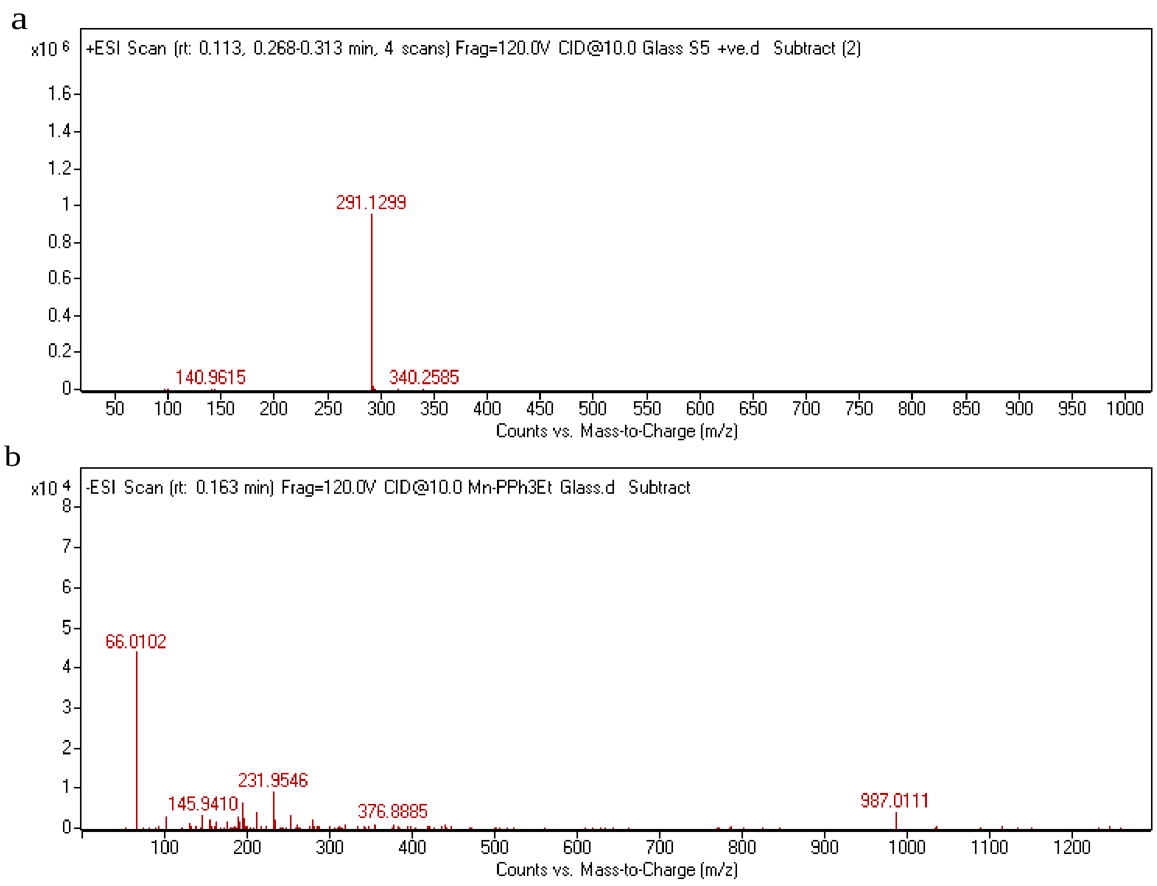

Figure S22: HRMS data of a_g(EtPh$_3$P)[Mn(dca)$_3$] with ionisation in (a) positive and (b) negative mode.

a

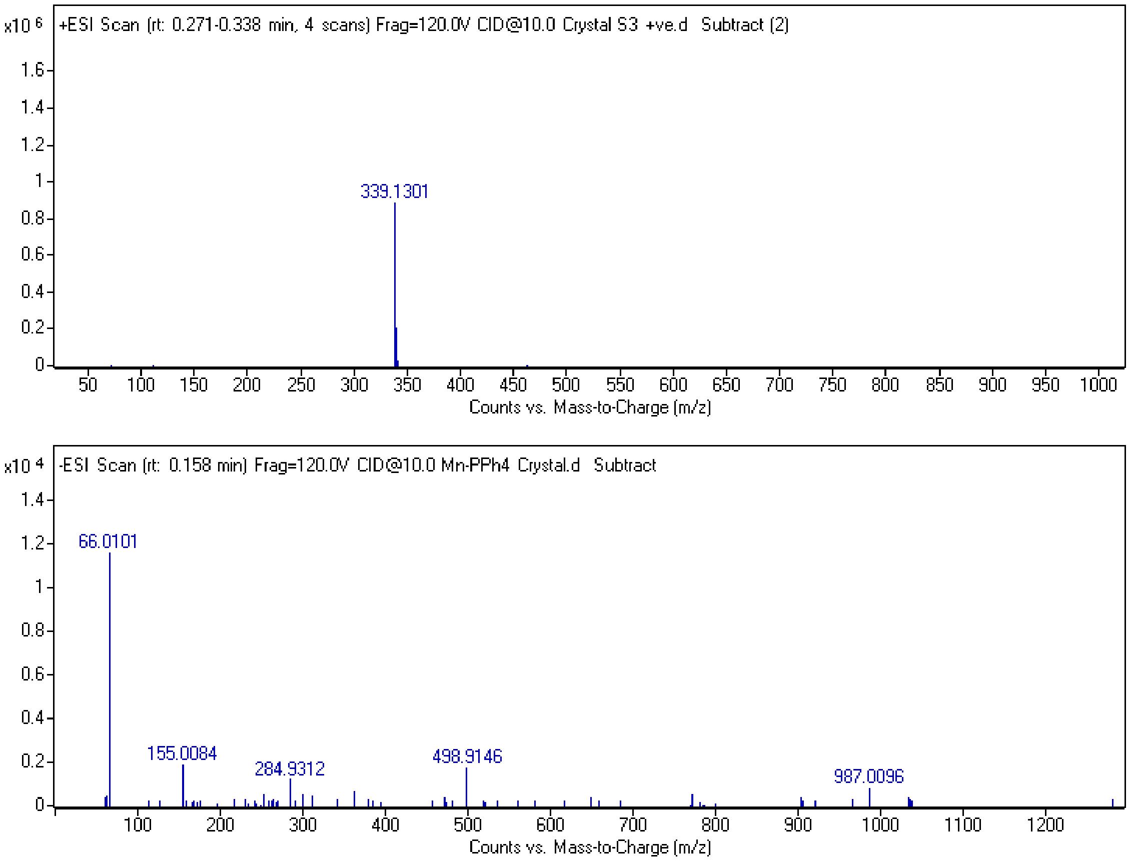

Figure S23: HRMS data of crystalline $(Ph_4P)[Mn(dca)_3]$ with ionisation in (a) positive and (b) negative mode.

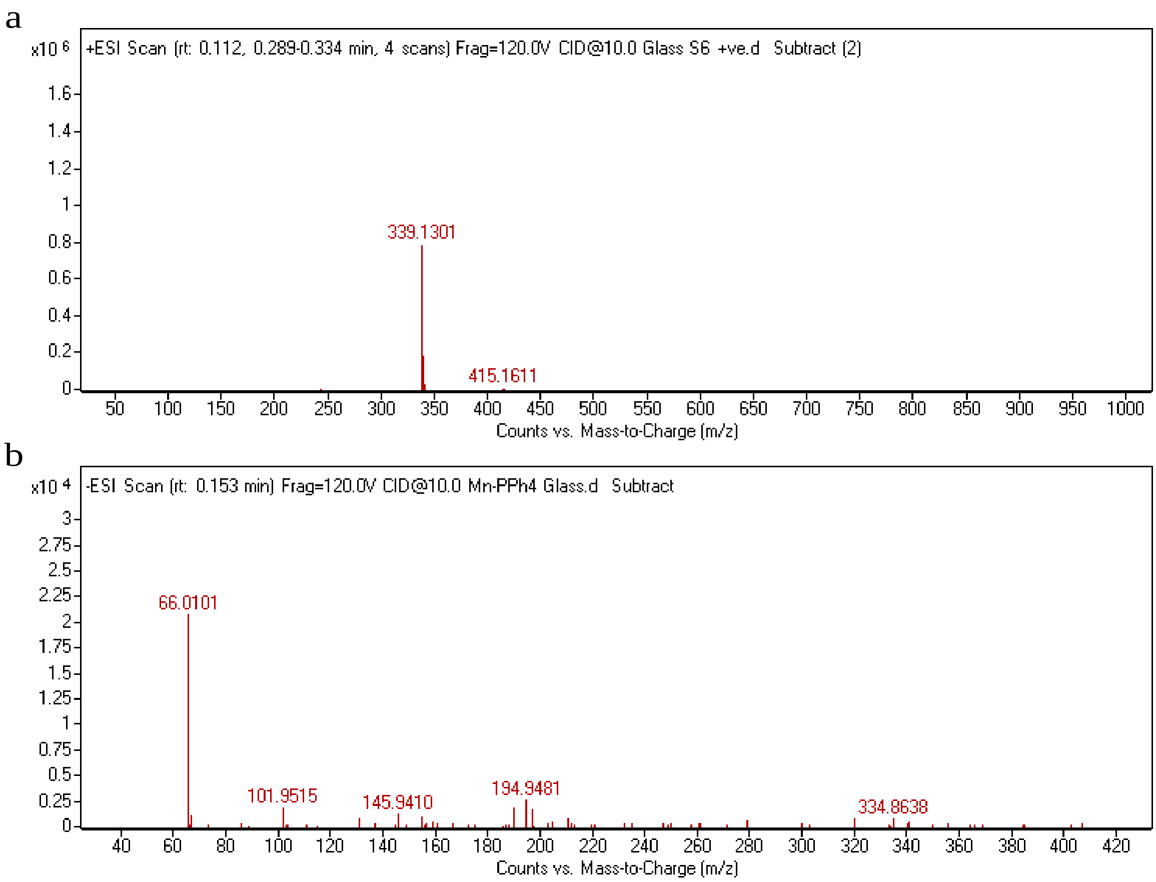

Figure S24: HRMS data of a_g(Ph_4P)[$Mn(dca)_3$] with ionisation in (a) positive and (b) negative mode.

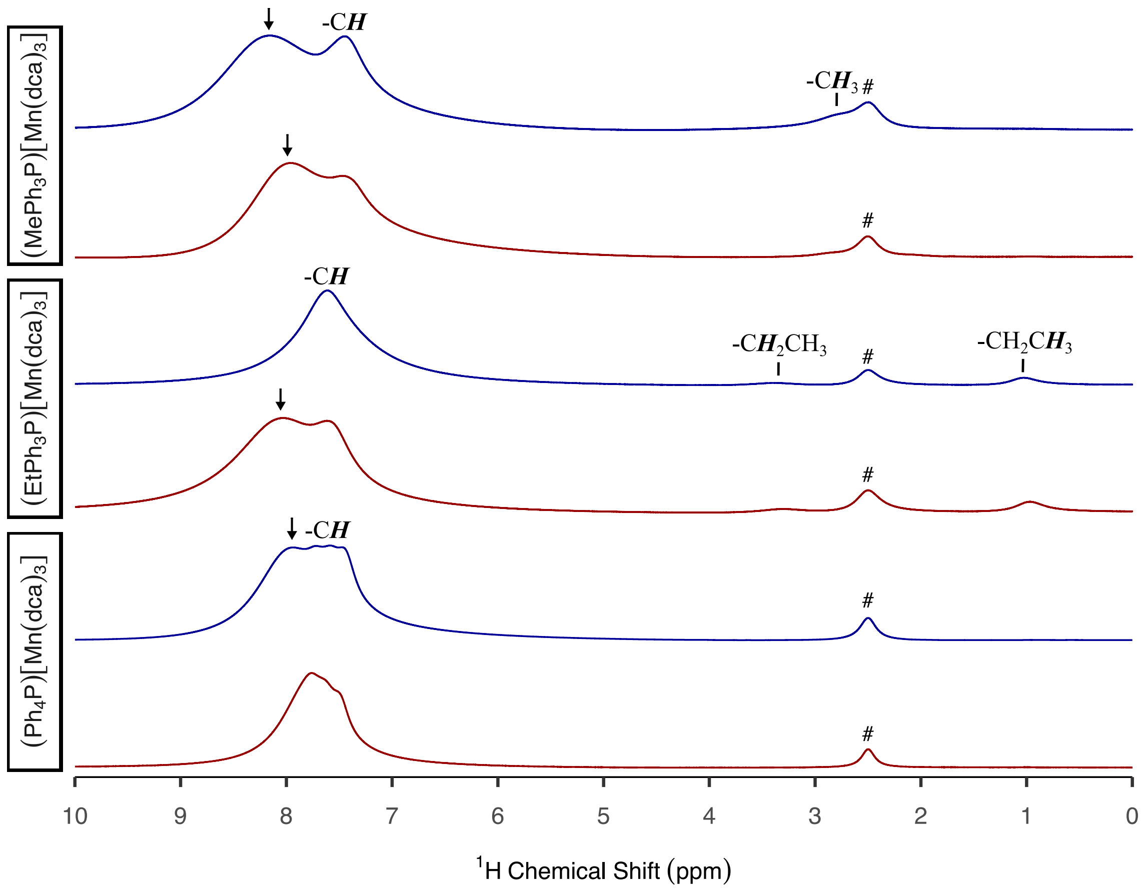

Figure S25: ^{1}H liquid-phase NMR spectra of (blue) crystalline $(RPh_3P)[Mn(dca)_3]$ (where R = Me, Et, Ph) and (red) $a_g(RPh_3P)[Mn(dca)_3]$. Hash signs (#) denote the ^{1}H signal corresponding to the residual protons in DMSO-d_6 at 2.50 ppm. The ^{1}H signals are assigned in the NMR spectra of the crystalline $(RPh_3P)[Mn(dca)_3]$ samples. The arrows denote ^{1}H signals that are tentatively assigned to an H_2O-protonated dca ligand which exists as an aminonitrile-carbodiimide tautomer in acidic conditions.

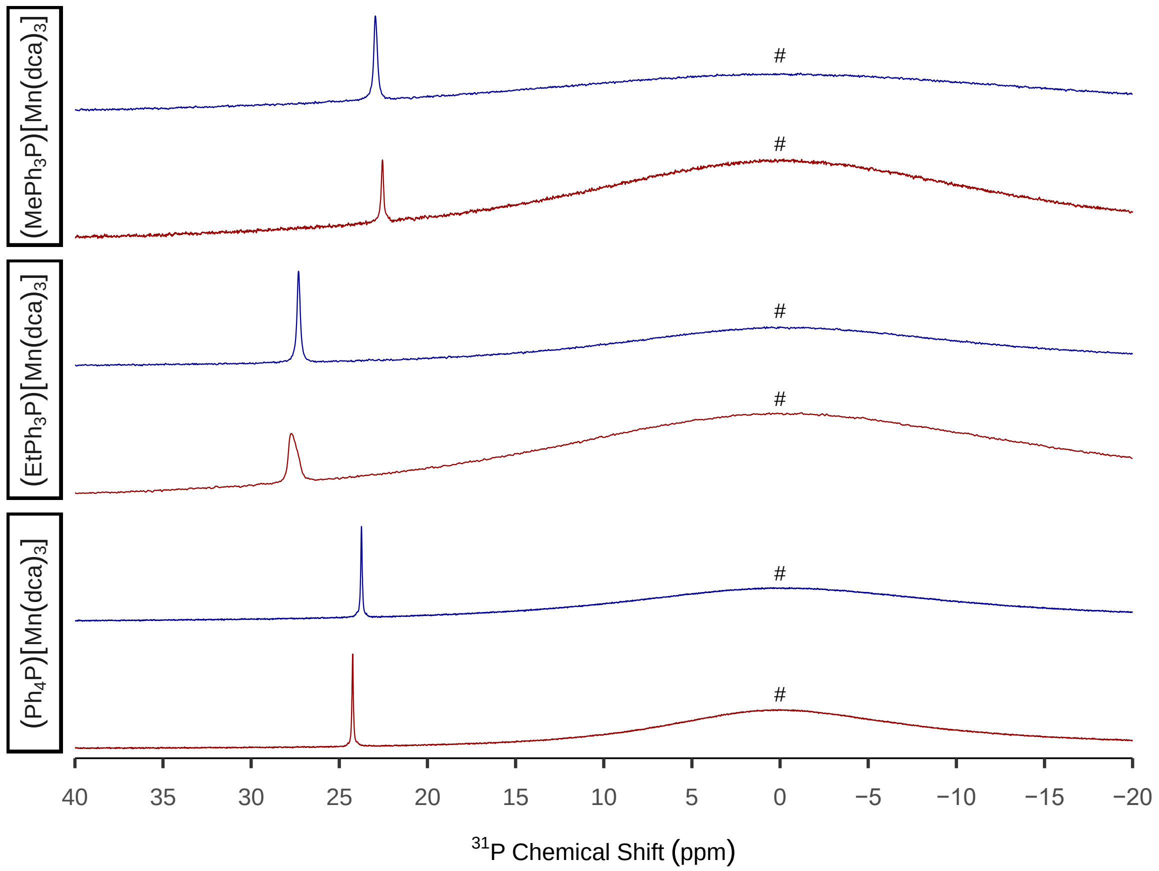

Figure S26: ^{31}P liquid-phase NMR spectra of (blue) crystalline $(RPh_3P)[Mn(dca)_3]$ (where R = Me, Et, Ph) and (red) $a_g(RPh_3P)[Mn(dca)_3]$. Hash signs (#) denote the ^{31}P H_3PO_4 signal at 0.00 ppm.

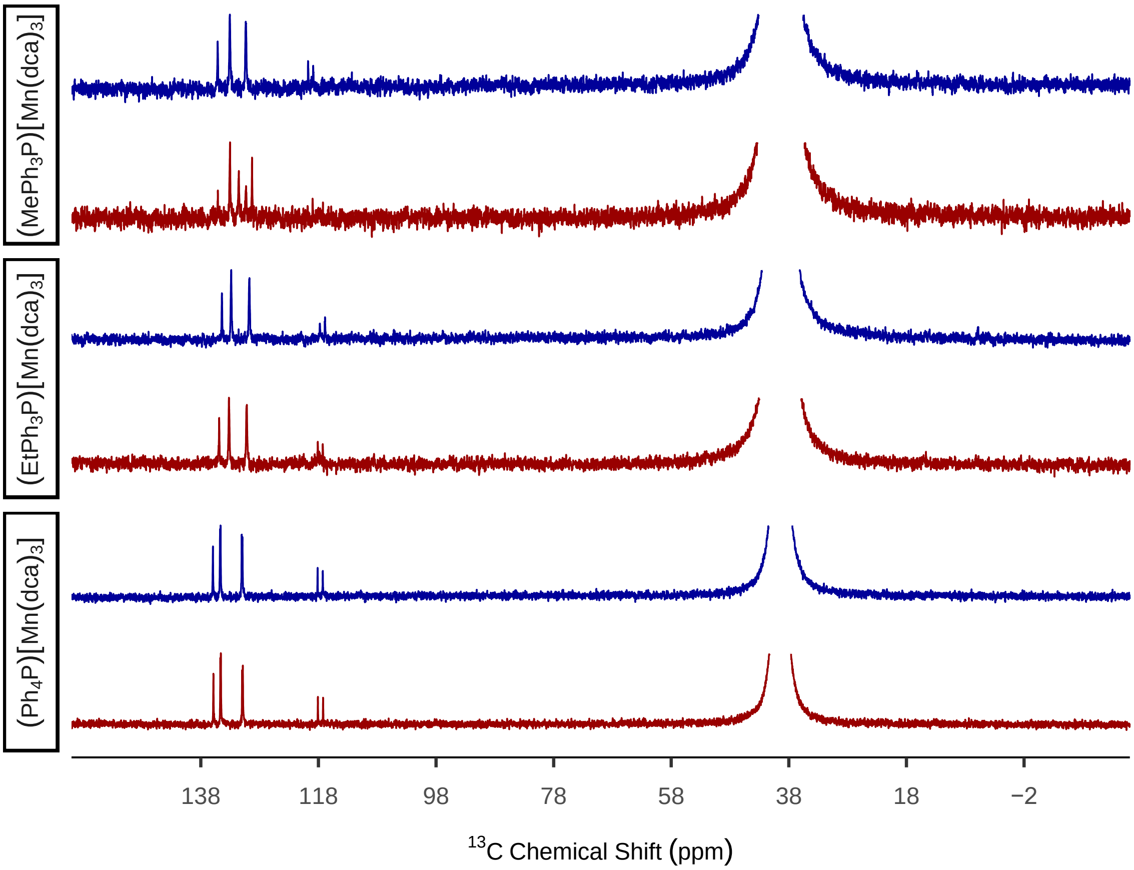

Figure S27: ^{13}C liquid-phase NMR spectra of (blue) crystalline (RPh_3P)[Mn(dca)$_3$] (where R = Me, Et, Ph) and (red) a_g(RPh_3P)[Mn(dca)$_3$]. The signal at 39.50 ppm corresponds to the DMSO-d$_6$ solvent.

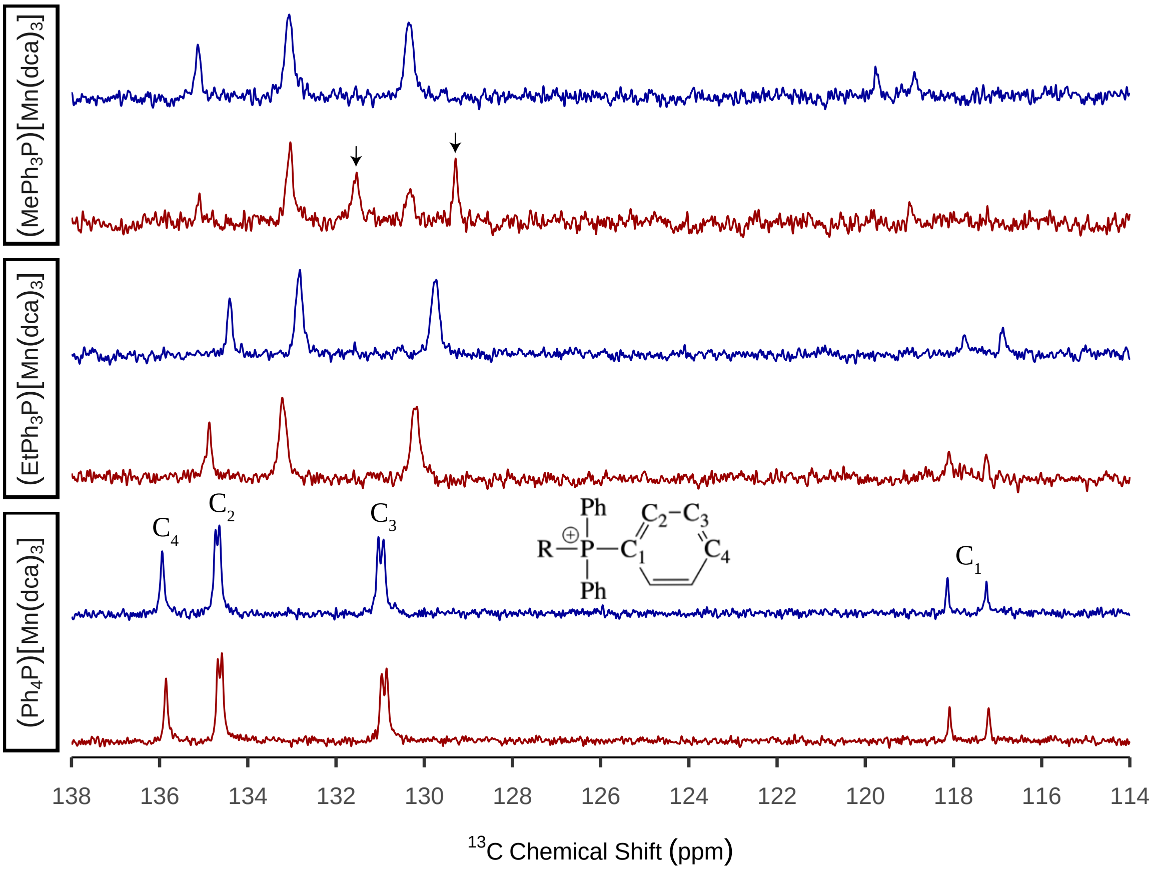

Figure S28: Magnified view of the 138 to 114 ppm region of the ^{13}C liquid-phase NMR spectra of (blue) crystalline $(RPh_3P)[Mn(dca)_3]$ (where R = Me, Et, Ph) and (red) $a_g(RPh_3P)[Mn(dca)_3]$. The signals marked with an arrow in the ^{13}C NMR spectrum of $a_g(MePh_3P)[Mn(dca)_3]$ are attributed to the poor solubility of the melt-quenched glass. The assignment of the ^{13}C signals is presented in the ^{13}C NMR spectrum of crystalline $(Ph_4P)[Mn(dca)_3]$, as this spectrum exhibits enhanced signal-to-noise ratio and resolution revealing ^{13}C-^{31}P scalar J coupling.

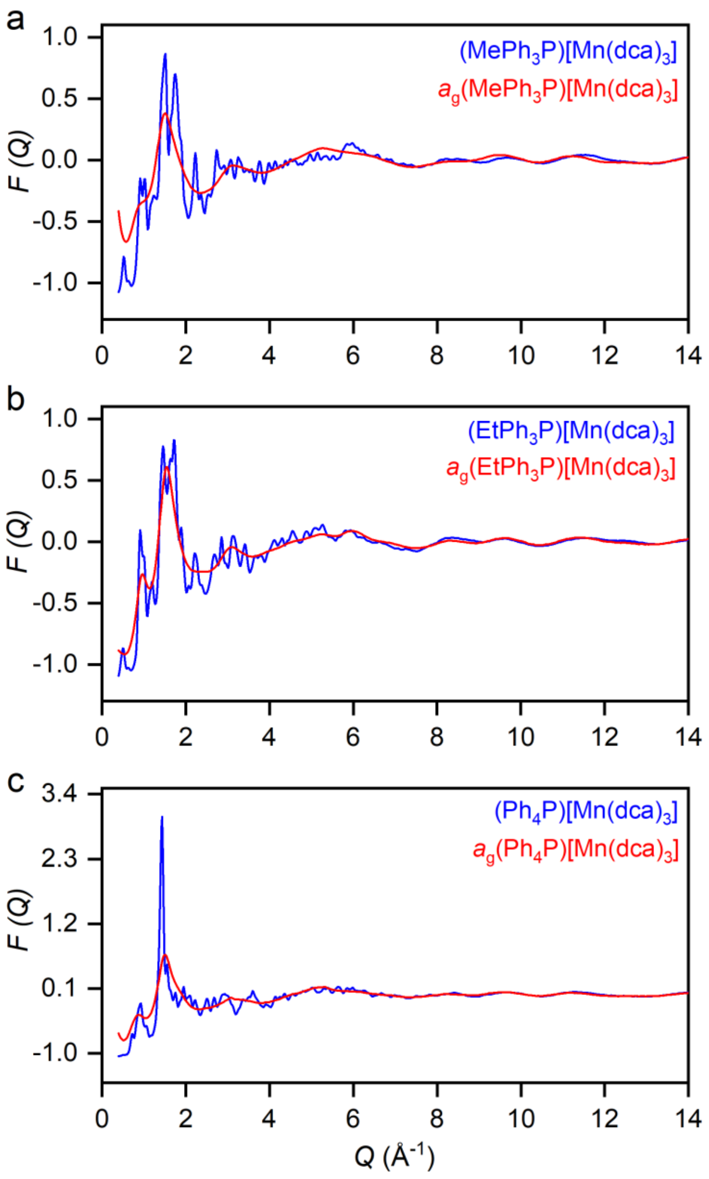

Figure S29: Room temperature X-ray total scattering data for $(RPh_3P)[Mn(dca)_3]$ and $a_g(RPh_3P)[Mn(dca)_3]$ (where R = Me, Et, Ph). Structure factors, $F(Q)$, of the samples before heating (blue - crystal) and upon melt-quenching (red - glass).

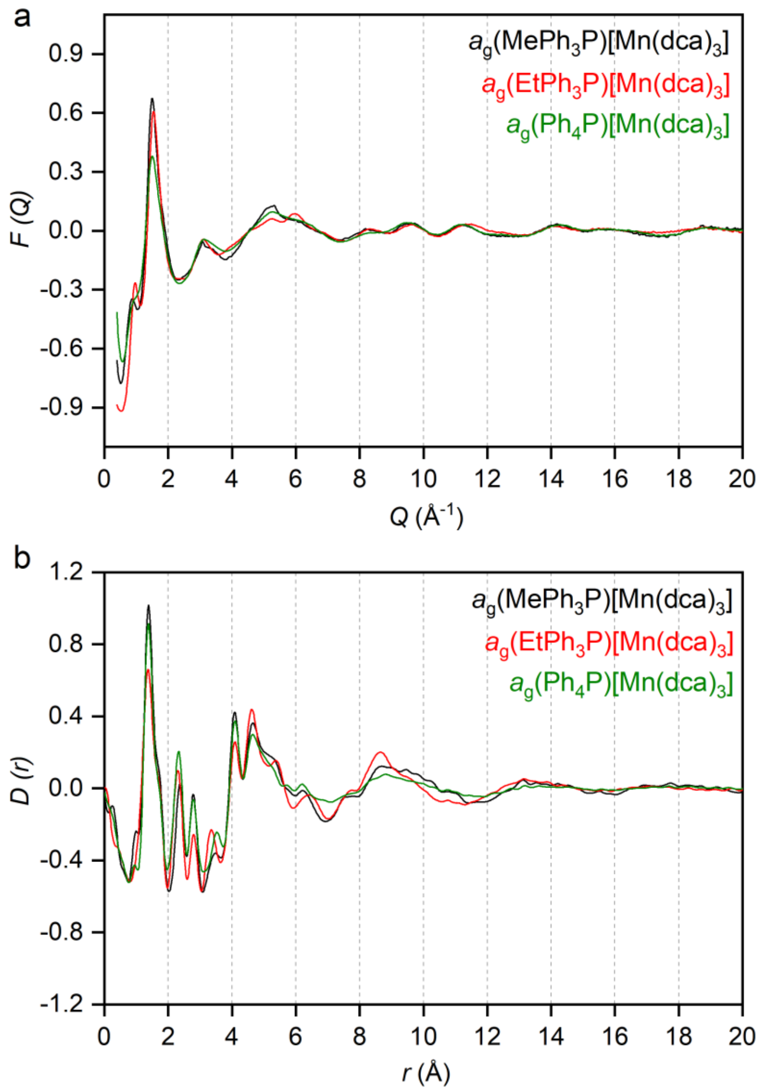

Figure S30: (**a**) Structure factors, $F(Q)$, and (**b**) Pair distribution functions, $D(r)$, for $a_g(RPh_3P)[Mn(dca)_3]$ (where R = Me, Et, Ph) glasses.

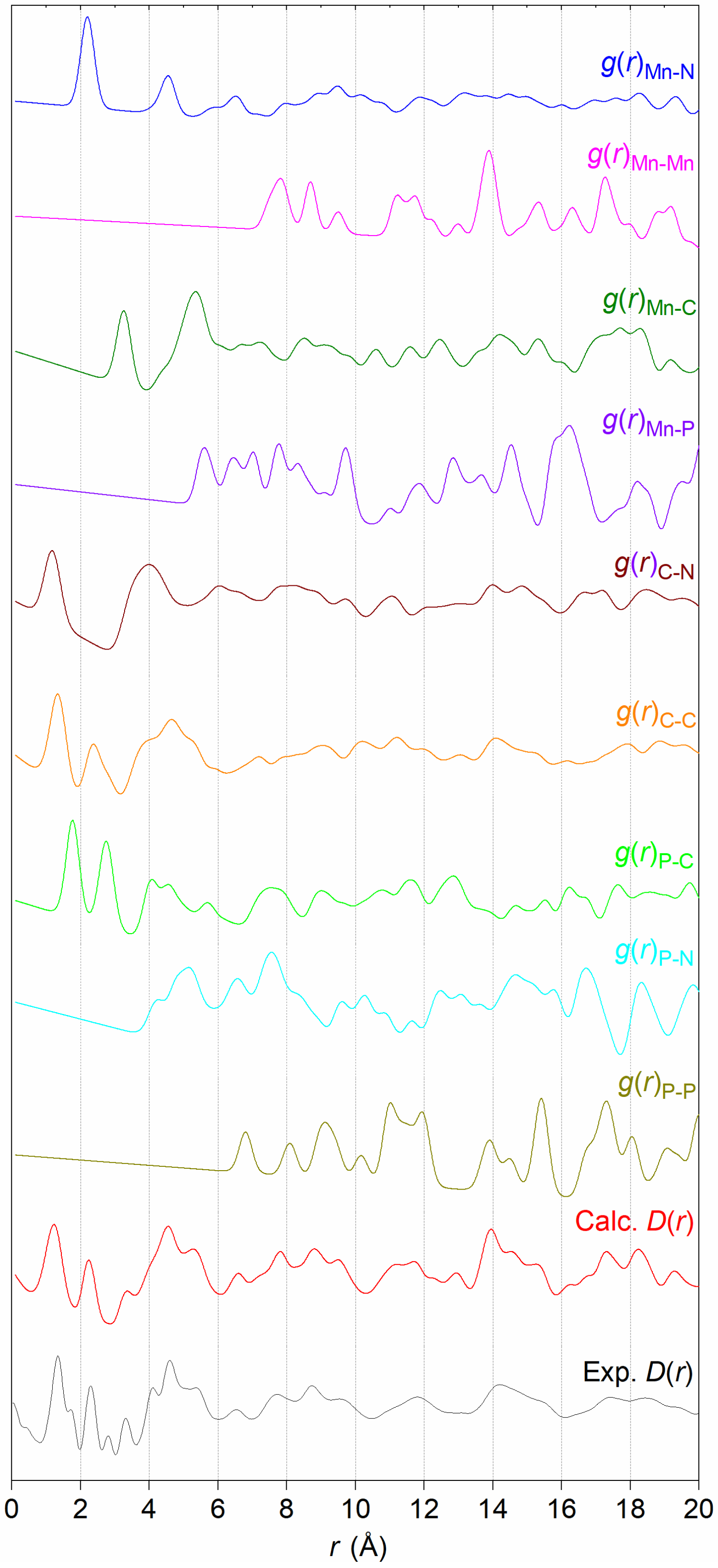

Figure S31: Comparison of calculated total pair distribution functions for $(MePh_3P)[Mn(dca)_3]$. These were calculated from PDFgui refinement of the published crystal structure.[1] Experimental $D(r)$ data is in black. Also shown are the calculated partial pair distribution functions $g_{ij}(r)$ that do not involve H.

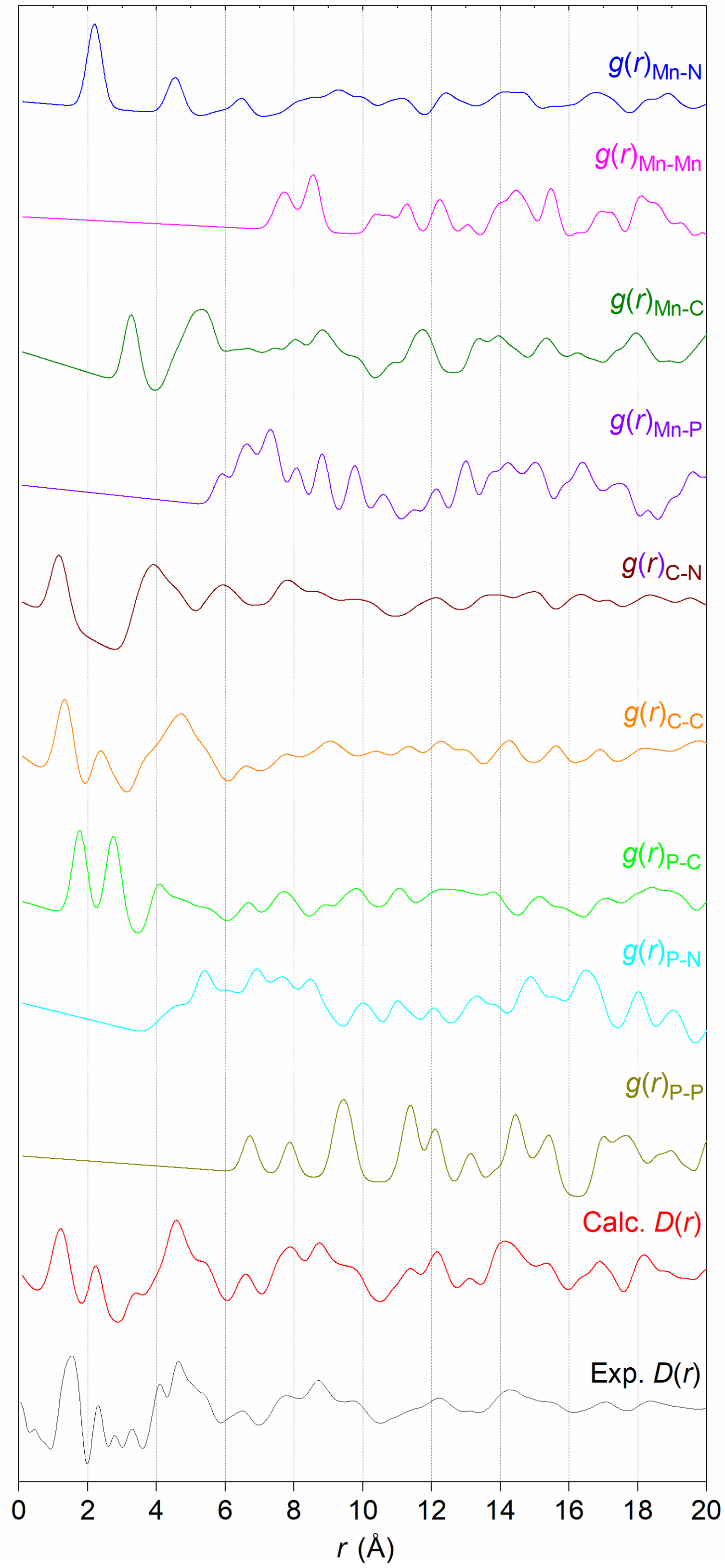

Figure S32: Comparison of calculated total pair distribution functions for $(EtPh_3P)[Mn(dca)_3]$. These were calculated from PDFgui refinement of the published crystal structure.[5] Experimental $D(r)$ data is in black. Also shown are the calculated partial pair distribution functions $g_{ij}(r)$ that do not involve H.

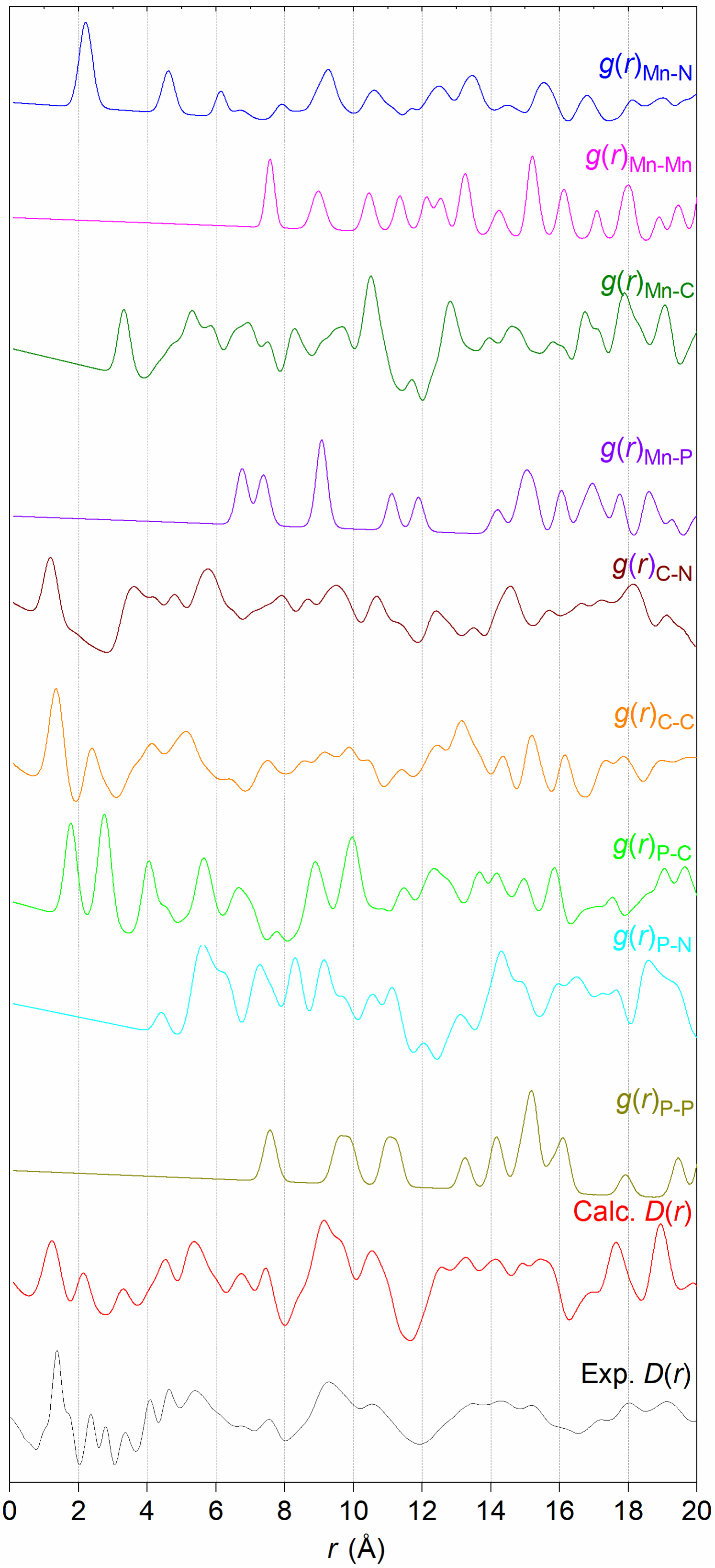

Figure S33: Comparison of calculated total pair distribution functions for $(Ph_4P)[Mn(dca)_3]$. These were calculated from PDFgui refinement of the published crystal structure.[1] Experimental $D(r)$ data is in black. Also shown are the calculated partial pair distribution functions $g_{ij}(r)$ that do not involve H.

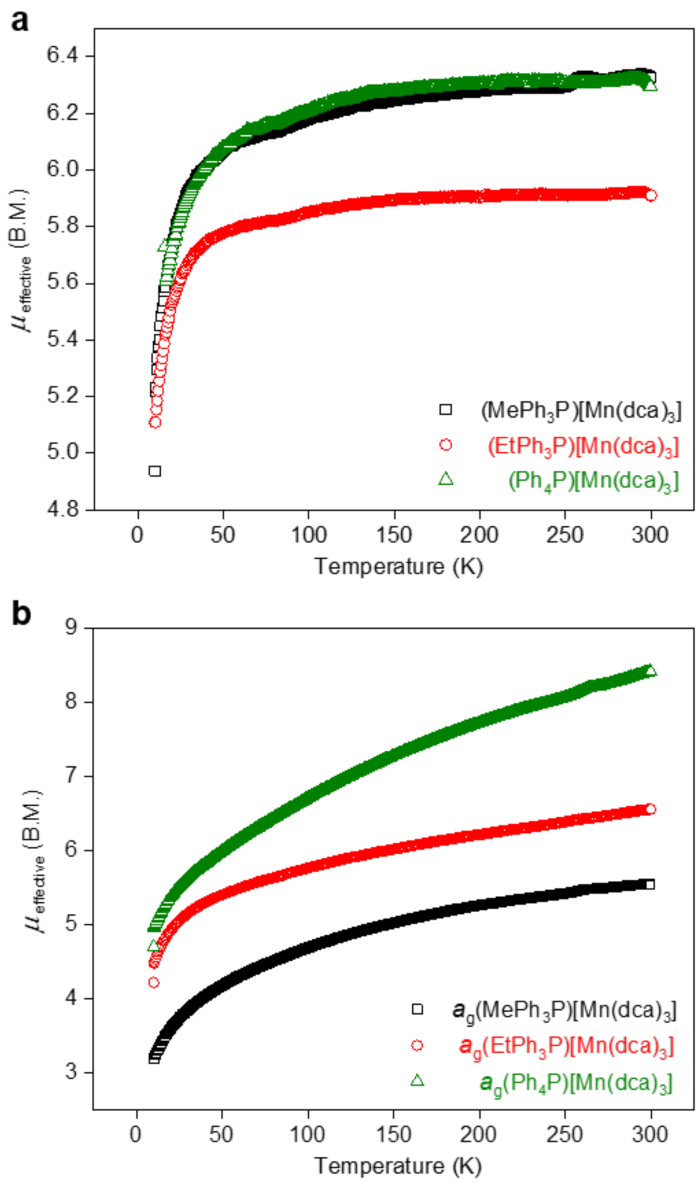

Figure S34: Variation of magnetic susceptibility in the form of effective magnetic moment (μ_{eff}) vs. temperature for (**a**) $(RPh_3P)[Mn(dca)_3]$ and (**b**) $a_g(RPh_3P)[Mn(dca)_3]$ samples (where R = Me, Et, Ph). A 100 Oe magnetic field was applied to measure the field-cooled susceptibility data.

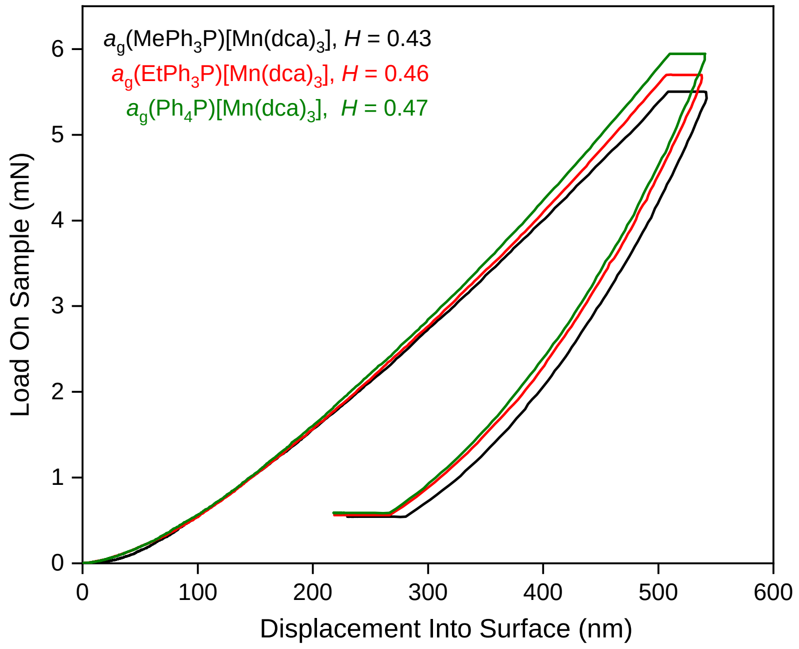

Figure S35. Nanoindentation experiments of the $a_g(RPh_3P)[Mn(dca)_3]$ samples (where R = Me, Et, Ph). Separate experiments of load are plotted against depth varied up to 500 nm.

Table S1. Comparison of values of the enthalpy of fusion (ΔH_f) and the entropy of fusion (ΔS_f) obtained after integrating the melting peaks both with baseline (shown in black dashed line in **Figure 2a**) or without baseline for $(MePh_3P)[Mn(dca)_3]$ compound with (TAlA)$[M(dca)_3]$ compounds.

**Samples**	**ΔH_f (without baseline) (kJ mol^{-1})**	**ΔS_f (without baseline) (J mol^{-1} K^{-1})**	**ΔH_f (with baseline) (kJ mol^{-1})**	**ΔS_f (with baseline) (J mol^{-1} K^{-1})**	**Ref**
$(MePh_3P)[Mn(dca)_3]$	50	98	52 [a]	101 [a]	This work
$(TPrA)[Fe(dca)_3]$	35	67	50 [a]	95 [a]	9
$(TPrA)[Co(dca)_3]$	49	101	65 [a]	134 [a]	9

[a] We have preferred the values in the manuscript obtained after integrating the peaks up to the melting offset temperature which defines the whole region of liquefaction succeeding with the parallel baseline alike its pre-melting state.

Table S2. Elemental composition (C, H, N, P) of $(RPh_3P)[Mn(dca)_3]$ (where R = Me, Et, Ph) structures in their crystalline and glassy (a_g) states.

**Samples**	**C**	**H**	**N**	**P**	**C-a_g**	**H-a_g**	**N-a_g**	**P-a_g**
$(MePh_3P)[Mn(dca)_3]$	56.48 (56.56)	3.25 (3.39)	23.35 (23.75)	6.29 (5.84)	52.62	3.27	21.75	6.24
$(EtPh_3P)[Mn(dca)_3]$	57.45 (57.31)	3.60 (3.67)	22.65 (23.14)	6.19 (5.69)	56.05	3.43	22.10	6.23
$(Ph_4P)[Mn(dca)_3]$	59.98 (60.76)	3.29 (3.37)	20.91 (21.26)	5.5 (5.22)	58.78	3.35	20.45	5.3

* Expected elemental compositions of the crystalline states were shown inside the first bracket.

Table S3. Pycnometric densities of $(RPh_3P)[Mn(dca)_3]$ (where R = Me, Et, Ph) structures in their crystalline (ρ_c) and glassy (ρ_g) states.

**Samples**	**ρ_c (g cm^{-3})**	**ρ_g (g cm^{-3})**
$(MePh_3P)[Mn(dca)_3]$	1.407 (±0.005)	1.425 (±0.003)
$(EtPh_3P)[Mn(dca)_3]$	1.376 (±0.003)	1.388 (±0.002)
$(Ph_4P)[Mn(dca)_3]$	1.392 (±0.004)	1.405 (±0.004)

References

(1) van der Werff, P. M.; Batten, S. R.; Jensen, P.; Moubaraki, B.; Murray, K. S. Cation Templation of Anionic Metal Dicyanamide Networks. *Inorg. Chem.* **2001**, *40* (7), 1718–1722.

(2) Shaw, B. K.; Castillo-Blas, C.; Thorne, M. F.; Ríos Gómez, M. L.; Forrest, T.; Lopez, M. D.; Chater, P. A.; McHugh, L. N.; Keen, D. A.; Bennett, T. D. Principles of Melting in Hybrid Organic–Inorganic Perovskite and Polymorphic ABX_3 Structures. *Chem. Sci.* **2022**, *13* (7), 2033–2042.

(3) Bain, G. A.; Berry, J. F. Diamagnetic Corrections and Pascal's Constants. *J. Chem. Educ.* **2008**, *85* (4), 532.

(4) Coelho, A. A. TOPAS and TOPAS-Academic : An Optimization Program Integrating Computer Algebra and Crystallographic Objects Written in C++. *J. Appl. Crystallogr.* **2018**, *51* (1), 210–218.

(5) van der Werff, P. M.; Batten, S. R.; Jensen, P.; Moubaraki, B.; Murray, K. S.; Cashion, J. D. Structure and Magnetism of 3D Anionic Metal Dicyanamide $(MePh_3P)[M(Dca)_3]$ (M = Fe, Co, Ni) and $(EtPh_3P)[M(Dca)_3]$ (M = Mn, Co, Ni) Networks. *Cryst. Growth Des.* **2004**, *4* (3), 503–508.

(6) Bermúdez-García, J. M.; Yáñez-Vilar, S.; García-Fernández, A.; Sánchez-Andújar, M.; Castro-García, S.; Mira, J.; Moreira, J. A.; Centeno, T. A.; Señarís-Rodríguez, M. A. A Simple in Situ Synthesis of Magnetic M@CNTs by Thermolysis of the Hybrid Perovskite $[TPrA][M(Dca)_3]$. *New J. Chem.* **2017**, *41* (8), 3124–3133.

(7) Kroke, E.; Schwarz, M.; Horath-Bordon, E.; Kroll, P.; Noll, B.; Norman, A. D. Tri-s-Triazine Derivatives. Part I. From Trichloro-Tri-s-Triazine to Graphitic C_3N_4 Structures. *New J. Chem.* **2002**, *26*, 508–512.

(8) Täuber, K.; Dani, A.; Yuan, J. Covalent Cross-Linking of Porous Poly(Ionic Liquid) Membrane via a Triazine Network. *ACS Macro Lett.* **2017**, *6*, 1–5.

(9) Shaw, B. K.; Hughes, A. R.; Ducamp, M.; Moss, S.; Debnath, A.; Sapnik, A. F.; Thorne, M. F.; McHugh, L. N.; Pugliese, A.; Keeble, D. S.; Chater, P.; Bermudez-Garcia, J. M.; Moya, X.; Saha, S. K.; Keen, D. A.; Coudert, F.-X.; Blanc, F.; Bennett, T. D. Melting of Hybrid Organic–Inorganic Perovskites. *Nat. Chem.* **2021**, *13*, 778−785.
